# Genetic diversity of *Leishmania donovani* that causes cutaneous leishmaniasis in Sri Lanka: a cross sectional study with regional comparisons

**DOI:** 10.1186/s12879-017-2883-x

**Published:** 2017-12-22

**Authors:** Udeshika Lakmini Kariyawasam, Angamuthu Selvapandiyan, Keshav Rai, Tasaduq Hussain Wani, Kavita Ahuja, Mizra Adil Beg, Hasitha Upendra Premathilake, Narayan Raj Bhattarai, Yamuna Deepani Siriwardena, Daibin Zhong, Guofa Zhou, Suman Rijal, Hira Nakhasi, Nadira D. Karunaweera

**Affiliations:** 1 Department of Parasitology, Faculty of Medicine, University of Colombo, No. 25, Kynsey Road, Colombo, 8 Sri Lanka; 2 JH-Institute of Molecular Medicine, Jamia Hamdard, New Delhi, India; 3 Department of Microbiology, B.P. Koirala Institute of Health Sciences, Dharan, Nepal; 4 Department of Biology, University of South Dakota, Vermillion, SD USA; 5 University of California Irvine, College of Health Sciences, Irvine, CA USA; 6 Division of Emerging and Transfusion Transmitted Diseases, Food and Drug Administration, Bethesda, USA

**Keywords:** Skin lesions, Antimony resistance, Visceral leishmaniasis, kDNA minicircle sequences, Haplotype diversity, Geographical clustering, Phenotypic characteristics, Nepal, India

## Abstract

**Background:**

*Leishmania donovani* is the etiological agent of visceral leishmaniasis (VL) in the Indian subcontinent. However, it is also known to cause cutaneous leishmaniasis (CL) in Sri Lanka. Sri Lankan *L. donovani* differs from other *L. donovani* strains, both at the molecular and biochemical level. To investigate the different species or strain-specific differences of *L. donovani* in Sri Lanka we evaluated sequence variation of the kinetoplastid DNA (kDNA).

**Methods:**

Parasites isolated from skin lesions of 34 CL patients and bone marrow aspirates from 4 VL patients were genotyped using the kDNA minicircle PCR analysis. A total of 301 minicircle sequences that included sequences from Sri Lanka, India, Nepal and six reference species of *Leishmania* were analyzed.

**Results:**

Haplotype diversity of Sri Lankan isolates were high (*H*_*d*_ = 0.757) with strong inter-geographical genetic differentiation (*F*_*ST*_ > 0.25). In this study, *L. donovani* isolates clustered according to their geographic origin, while Sri Lankan isolates formed a separate cluster and were clearly distinct from other *Leishmania* species. Within the Sri Lankan group, there were three distinct sub-clusters formed, from CL patients who responded to standard antimony therapy, CL patients who responded poorly to antimony therapy and from VL patients. There was no specific clustering of sequences based on geographical origin within Sri Lanka.

**Conclusion:**

This study reveals high levels of haplotype diversity of *L. donovani* in Sri Lanka with a distinct genetic association with clinically relevant phenotypic characteristics. The use of genetic tools to identify clinically relevant features of *Leishmania* parasites has important therapeutic implications for leishmaniasis.

**Electronic supplementary material:**

The online version of this article (10.1186/s12879-017-2883-x) contains supplementary material, which is available to authorized users.

## Background

Leishmaniases, caused by the *Leishmania* species, are a group of vector-borne parasitic diseases with a high disease burden in the Indian sub-continent [1, 2]. The main clinical forms are cutaneous leishmaniasis (CL), muco-cutaneous leishmaniasis (MCL) and visceral leishmaniasis (VL), which are caused by different species and subspecies of *Leishmania* [1, 3]*. Leishmania donovani* is widely recognized to cause VL in the old world; while in the new world VL is caused by a closely related species, *L. infantum* [3–6]*.* The causative agent of CL in Sri Lanka is *L. donovani* zymodeme MON-37 [7, 8], which is closely related to the common VL causing zymodeme MON-2 in India [9].

CL is now considered as an endemic disease in Sri Lanka, with a steady increase in the number of cases detected [10, 11]. Since 2001, more than 6500 cases of CL [12] have been reported along with a few MCL and VL cases [13–15]. The steady increase of CL in Sri Lanka is complicated by the appearance and spread of CL that does not respond to the standard antimonial drugs routinely used in clinical practice [16]. Initial studies in Sri Lanka have indicated that both CL and VL are caused by *L. donovani-* MON-37 [7, 15]. However, the existence and contribution of intra-species variation that influences the clinical manifestations of leishmaniasis and the treatment response to antimonial drugs in Sri Lanka is unknown.

*Leishmania donovani* virulence factors involved in pathogenesis, visceral organ invasion and parasite persistence, have been extensively studied over several decades [17–19]. The A2 protein production [18, 20, 21], lipophosphoglycan (LPG) activity [22–24], glycoprotein 63 (gP63) gene expression [25, 26], acid phosphatase activity [18, 27] and variations in the mini-exon genes of chromosome 36 [17] are known factors that influence the virulence of *Leishmania* parasites. Further analysis of Sri Lankan *L. donovani* isolates is thus warranted in order to gain a better understanding of differences in virulence and pathogenicity.

The genetic characterization of *Leishmania* parasites can be achieved via the analysis of several genetic markers such as mini-exons, microsatellites, ribosomal internal transcribed spacer (ITS) regions and minicircles of kinetoplast DNA [28–31]. The kinetoplast DNA (kDNA) network in *Leishmania* is composed of two types of DNA rings; maxicircles and minicircles. The *Leishmania* parasite minicircle kDNA is approximately 1 kb in size, containing a conserved region that is ~200 bp in length and a variable region of ~600 bp. *Leishmania* minicircle kDNA provides an ideal target for the genotyping of *Leishmania* parasite as the sequence differences allow for accurate discrimination between species [32–34].

Previous studies using the minicircle kDNA footprint assay have enabled rapid identification of known or unknown species with a high level of sensitivity [35]. This method is considered to be better suited as compared to other frequently used speciation methods, such as Restriction Fragment Length Polymorphism (RFLP) and Southern blot which depend on low-copy PCR targets (non–minicircle DNA regions) and have limitations in *Leishmania* with regard to species identification [35–37].

The aim of this study was to use the kDNA footprint assay to study *L. donovani* strain specific sequence diversity and determine the association between sequence variations and distinct clinical characteristics in individuals with leishmaniasis in Sri Lanka.

## Methods

### Study setting and study population

#### Sri Lanka

**Fig. 1:**
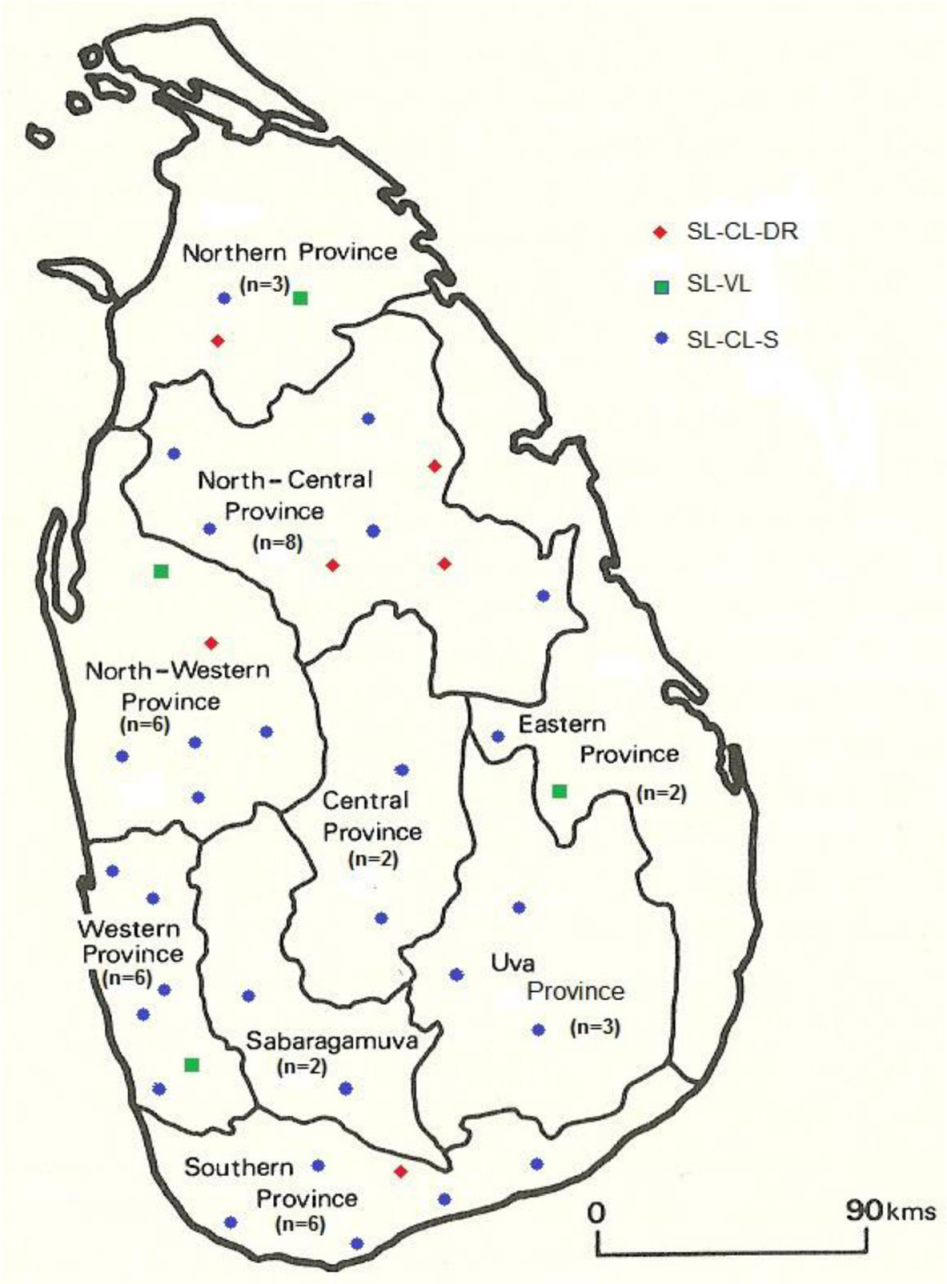
Geographical distribution of leishmaniasis patients in Sri Lanka included in the study. The geographical origin of leishmaniasis patients within the main administrative provinces of the country are shown () with the number of patients from each province given within parenthesis. SL-CL-S: patients with uncomplicated cutaneous leishmaniasis, SL-CL-DR: patients with cutaneous leishmaniasis that showed poor response to antimony therapy and SL-VL: visceral leishmaniasis patients

A cross-sectional study of confirmed clinical cases was carried out to represent all the administrative provinces of Sri Lanka over a one year period (Fig. 1). Study participants who were laboratory-confirmed as either CL (*n* = 34) or VL (*n* = 4) were selected. Patients with other skin diseases and who were negative for laboratory diagnosis were excluded. Within the CL group, there were six patients (*n* = 6), who had a history of poor response to the routine local treatment of intra-lesional sodium stibogluconate (IL-SSG). A patient was considered a ‘poor responder’, if the lesion size (ulceration area in the cases of ulcerative lesions or the induration area in the cases of non-ulcerative lesions) did not decreased by a minimum of 50% of the pre-treatment size, as judged by the collaborating dermatologists, following a minimum of 10 IL-SSG injections given at weekly intervals, similar to previously described criteria [38–40].

#### Nepal and India

Parasite DNA extracts collected via archived samples of *L. donovani* parasites (from skin lesions of post kala-azar dermal leishmaniasis (PKDL) patients and bone marrow of VL patients) maintained at collaborative centers in India (*n* = 27) and Nepal (*n* = 26), were used for comparative experiments. The kDNA sequences from clinical isolates stored in public databases (GenBank) were also included in the analyses.

### Laboratory confirmation of leishmaniasis

Prior to inclusion in the study, diagnosis of infection was confirmed in all study subjects by the demonstration of *Leishmania* parasites by oil immersion (X1000) microscopy of Giemsa-stained smears of lesion aspirates or slit-skin scrapings of CL patients and bone marrow aspirates of VL patients, either directly or following in-vitro culture. Parasites were grown in M199 medium (Gibco, Invitrogen, USA) supplemented with 15% heat inactivated fetal bovine serum (FBS) (Gibco, Invitrogen, USA) with Penicillin-Streptomycin (Sigma-Aldrich, USA) and the cultures were maintained at 26 °C. Cultured parasites were used for DNA extraction with the use of QIAamp DNA minikit (Qiagen, USA). Laboratory-confirmed patients were referred to the local dermatologist (for CL) and/or consultant physicians (for VL) for treatment according to Sri Lankan Ministry of Health guidelines. IL-SSG poor-responders in the CL group were those re-referred to the department for repeat investigations due to delay or absence of clinical cure.

### DNA preparation and sequencing

Extracted DNA was subjected to a fluorescence-based PCR followed by cloning and sequencing of the resulting DNA fragment according to established protocols [34]. The PCR reaction for kDNA amplification was performed using primers from conserved regions across kinetoplast minicircles of *Leishmania spp*. (primer JW11–5’CCTATTTTACACCAACCCCCAGT3’ and primer JW12–5’GGGTAGGGGCGTTCTGCGAAA3’) which generate an approximately 120 bp amplicon [34, 41–43]. Subsequently the amplified products were ligated in the pGEM-T vector and the resulting ligation products were transformed in to *E. coli* TOP 10 competent cells and plated for blue/white screening on plates containing ampicillin (100 μg/ml), X-gal (20 mg/ml) and iso-propyl-thio-galactoside (10 μl/ml of 100 mM). The resulting white colonies indicated transformed cells and blue colonies represented non-transformed ones. Individual white transformed colonies were used for plasmid DNA preparation followed by restriction digestion of 1 μg of plasmids using *EcoRI* enzyme at 37 °C (Promega, USA), according to the manufacturer’s recommendations. The total reaction volume was subjected to electrophoresis, in 2% agarose gel to confirm the presence of an insert. The inserts from the individual clones were sequenced with M13 forward primer.

### Genetic analysis

#### Minicircle sequence analysis of parasite isolates

A total of 301 *L. donovani* sequences of parasite isolates from Sri Lanka (*n* = 38), India (*n* = 27) and Nepal (*n* = 26) were analyzed, along with minicircle sequences of 6 *Leishmania* reference species from different countries [34]. Multiple sequence alignment (MSA) was done, via CLUSTAL-X MSA Program, version 2.0, with a gap opening penalty of 10.00; gap extension penalty of 0.05; DNA transition weight of 0.50 [44, 45]. The DnaSP software was utilized to measure haplotype diversity (*H*_*d*_), Nucleotide diversity (*N*_d_) and pair wise fixation index (*F*_*ST*_) for each group [46]. Furthermore, Tajima’s *D* Test [47] and Fu’s *F*s test [48] were also performed for detection of population growth (neutrality test for selection). The Haplotype diversity is defined as *H*_*d*_ = [*n*/(n − 1)] [1 − Σ *P*_*i*_^*2*^], with *n* being the number of sequences analyzed and *p*_*i*_ is the relative frequency of haplotype *i*. The nucleotide diversity is defined as, *N*_*d*_ = [*n*/(n − 1)] [1 − Σ *x*_*ij*_^*2*^], where *x*_*ij*_ is the relative frequency of nucleotide variant *j* (*j* = 1, 2, 3 and 4 correspond to A, C, G and T) at site *i*. *H*_*d*_ > 0.5 and *N*_*d*_ > 0.1 reflect high haplotype and nucleotide diversity, respectively within the study population [46, 49].

Tajima’s D Test is a measure of selection through the patterns of genetic diversity within populations, whether they are consistent with neutral expectations. Positive D value suggests a recent population bottleneck or balancing selection, while negative D value suggests population expansion or purifying selection [50]. Fu’s Fs test is another measure of selection, which is based on the infinite sites model of mutation, and tests the number of alleles in a population and indicates whether they are consistent with neutral expectations. Fu’s statistic is thought to be a more sensitive indicator of population expansion and genetic hitchhiking than Tajimas D. *F*_*ST*_ measures genetic differentiation among subpopulations. According to established standards, *F*_*ST*_ values from 0.0–0.05 indicate little genetic differentiation, whereas values that range from 0.05–0.15, 0.15–0.25 and >0.25 are considered to be representing moderate, great and strong genetic differentiation, respectively [51, 52]. The ‘isolation by distance analysis’ was performed using IBDWS [53]. Three populations were defined for this analysis viz., Sri Lanka, India and Nepal.

#### Phylogenetic analysis

Phylogenetic analysis was done using all sequences (*n* = 301) generated as described above. Previously published minicircle sequences of *L. donovani* (DD8), *L. infantum* (TK), *L.infantum* (SP), *L. major* (Al), *L. braziliensis* (P), *L.tropica (*TK*)* and *L. amazonensis* from the minicircle sequence database of GenBank, were also used in the phylogenetic analysis as reference sequences. The phylogenetic trees were constructed using the neighbor joining method, implemented in MEGA7.0.7 [54]. The evolutionary distances were computed using the Maximum Composite Likelihood method and depicted in units of number of base substitutions per site [54–56]. The tree was drawn to scale, with branch lengths in the same units as those of the evolutionary distances used to infer the phylogenetic tree. The resulting Guide Trees, showed cluster relationships among the sequences and allowed the assignment of test sequences to one of the reference species.

## Results

PCR amplification of *Leishmania* kDNA sequence segments from Sri Lanka showed a single approximately 120-bp band on 2% agarose gel, which was consistent with the product size of *L. donovani* as shown previously [34]. The multiple sequence alignment clearly showed a high level of genetic variation within the variable regions, whereas the conserved regions showed 83% nucleotide identity on average, in all sequences (Additional file 1).

The genetic diversity among Sri Lankan sequences was high, evidenced by the high haplotype diversity (*H*_*d*_ = 0.757) and high nucleotide diversity (*N*_*d*_ = 0.03) that were, comparable to the values of other tested *L. donovani* sequences from the Indian subcontinent (Table 1). Out of the *H*_*d*_ values generated for the Indian subcontinent, the highest value (*H*_*d*_ = 0.889) was observed for the Indian parasite sequences, with similar trends seen with nucleotide diversity (*N*_*d*_ = 0.093) (Table 1). Negative values were observed for both Fu’s Fs test (−25.801) and Tajima’s D test (−1.098) for Sri Lankan sequences only (Table 1). In contrast, positive Fu’s Fs test and Tajima’s D values were observed for sequences from India and Nepal.

**Table 1:** Genetic diversity parameters of *Leishmania donovani* isolates from Sri Lanka, India and Nepal

Origin	Sri Lanka	India	Nepal
Haplotype diversity (*H*_*d*_)	0.757	0.889	0.725
Nucleotide diversity (*N*_*d*_)	0.034	0.093	0.128
Number of sequences used (n)	166	51	52
Number of polymorphic sites(S)	33	36	34
Number of Haplotypes (h)	43	8	5
Tajima’s *D* (*P*-value)	−1.098(>0.1)	1.197(>0.1)	2.646(<0.01)
Fu’s *F*s tests(*P*-value)	−25.801 (>0.02)	10.780(<0.02)	21.900(<0.02)

Fu’s Fs: Not significant, *P* > 0.02. Tajima’s D: Not significant, *P* > 0.10

Inter geographical genetic differentiation (*F*_*ST*_) of *Leishmania* parasite populations from Sri Lanka, India and Nepal is depicted in Table 2. There was significant (*P* < 0.05) genetic differentiation of Sri Lankan *Leishmania* sequences when compared with those that originated from other countries included in the analysis (Table 2). Significantly high levels of genetic differentiation (*F*_ST_ > 0.25) was observed between Sri Lankan sequences and those from India and Nepal, while there was moderate levels of differentiation (<0.25) between Indian sequences and those from Nepal (Table 2). The isolation by distance analysis showed a linear correlation between genetic diversity and geographic distance, which might demonstrate the effects of gene flow or genetic barrier among parasite populations (Additional file 2). However, this correlation was not statistically significant.

**Table 2:** kDNA based genetic distances (*F*_*ST*_) between populations of *Leishmania* parasites from selected geographical areas

	Sri Lanka	India	Nepal
Sri Lanka	0.000		
India	0.347	0.000	
Nepal	0.522^a^	0.236	0.000

^a^Significant at level of 0.05

Diagonal: genetic distance within major geographic areas; below diagonal: genetic distance between populations

**Fig. 2:**
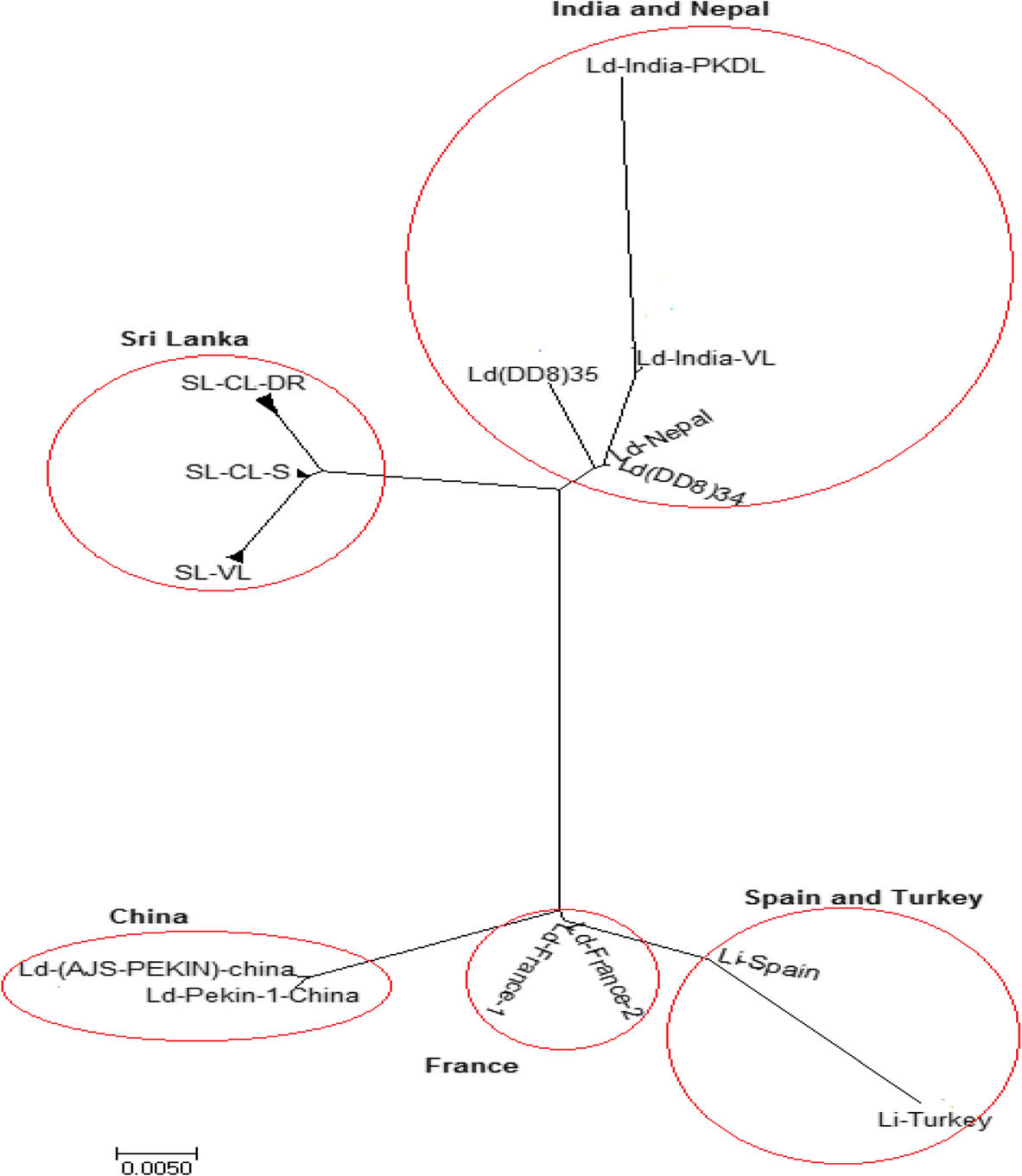
Phylogenetic tree constructed with the sequences of *L. donovani* isolates from Sri Lanka and other geographic locations. Ld-India-VL:*L. donovani* [India] (Bone marrow);Ld(DD8):*L. donovani* [India] (Bone marrow); SL-VL: *Leishmania* VL Sri Lankan isolates (Bone marrow); SL-CL-S:*Leishmania* CL Sri Lankan isolates (Skin); SL-CL-DR: *Leishmania* CL Sri Lankan isolates not respond to drug(Skin);Ld-Nepal: *L.donovani*[Nepal](Bone marrow); Li-Turkey: *L. infantum*[Turkey] (Spleen); Li-Spain:*L*. *infantum* [Spain] (Unknown);Ld-Peakin-China: *L. donovani*[China];Ld-France: *L. donovani* [France]

**Fig. 3:**
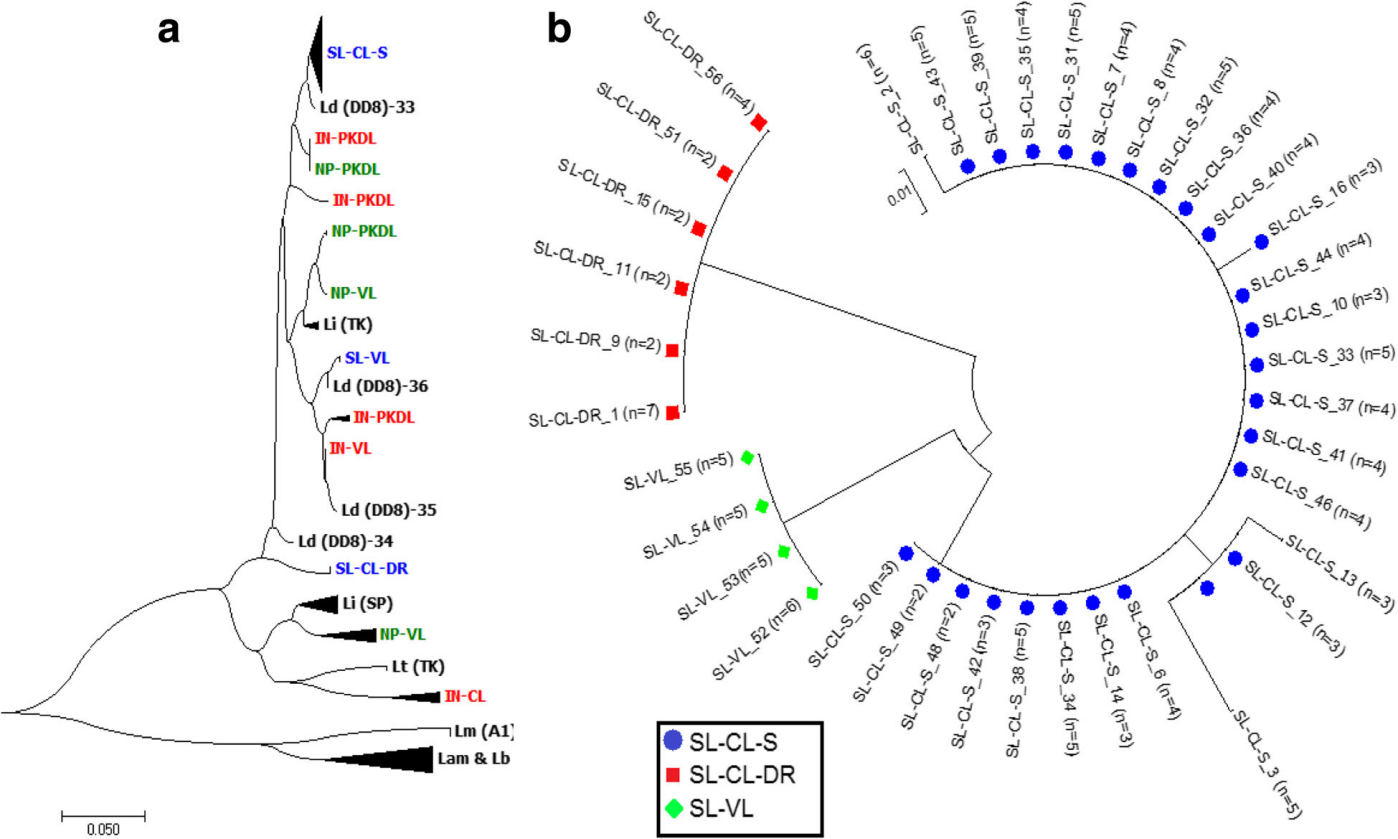
Phylogenetic trees with the *Leishmania* strains from Sri Lanka (SL), India (IN) and Nepal (NP) with (**a**) and /without (**b**) the global *Leishmania* reference panel consisting of different species of Leishmania from different regions. Lb(P):*L. braziliensis* [Peru] (Skin);Ld(DD8)/Ld(IN):*L. donovani* [India] (Bone marrow); Li(SP):*L. infantum* [Spain] (Unknown); Lm(A1):*L. major* [Libya]; Lt(TK):*L. tropica* [Turkey] (Skin); Lam: *L. amazonenis* (Skin); Li(TK):*L. infantum* [Turkey] (Spleen); SL-VL (52–55): VL Sri Lankan isolate (Bone marrow); SL_CL-S (2,3,6–8,10–14,16,17,31–46,48–50):*Leishmania* CL Sri Lankan isolate (Skin); SL_CL-DR (1,9,11,15,51,56):*Leishmania* CL Sri Lankan that failed to respond to antimonial drugs (Skin); IN-VL: *L. donovani* [India] (Bone marrow); IN-PKDL: *Leishmania* from post-kala azar dermal leishmaniasis (PKDL) [India] (Skin); NP-VL: *Leishmania* VL Nepalian isolate (Bone marrow); NP-PKDL:*Leishmania* cause PKDL [Nepal] (Skin)

In the phylogenetic analysis, all *Leishmania* sequences from Sri Lanka appeared to form a uniquely distinguishable group and were clearly distinct from other new and old world cutaneous leishmaniasis species found elsewhere (Figs. 2 and 3a). However, the Sri Lankan minicircle sequences were better linked in a grouping with other visceralizing *Leishmania* parasites, rather than the dermotropic species (Fig. 3a). Furthermore, the sequences from Sri Lanka, India and Nepal formed sister clusters when analyzed with other *Leishmania* sequences from the global reference panel (Fig. 3a). All the sequences from Sri Lanka were grouped alongside *L. donovani* (Fig. 3a). Within the Sri Lankan group, clear clustering was apparent among parasites that belonged to different clinical groups, which included uncomplicated cutaneous leishmaniasis (SL-CL-S), cutaneous leishmaniasis that showed poor response to antimony therapy (SL-CL-DR) and visceral disease (SL-VL) (Fig. 3a and b).

**Fig. 4:**
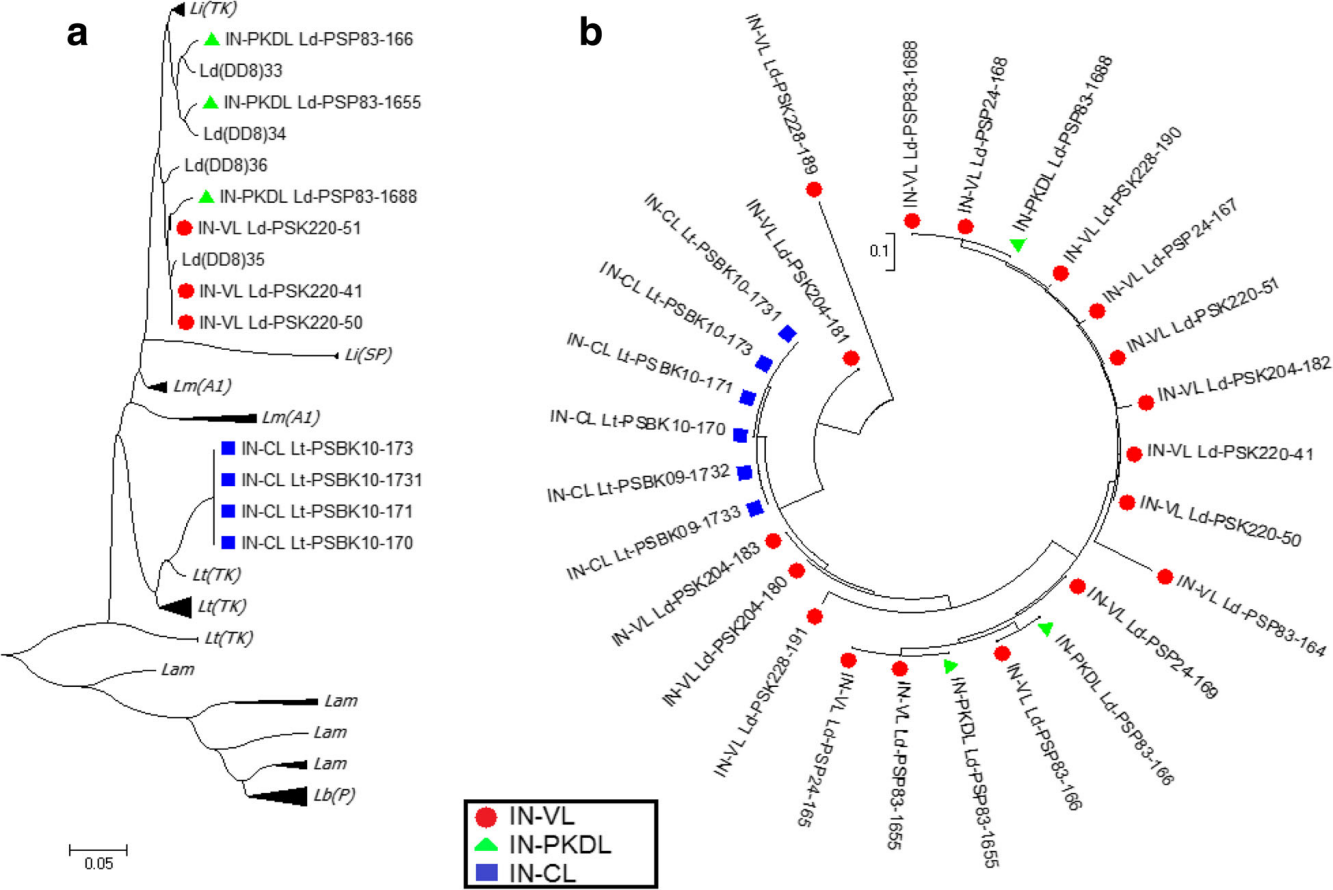
Phylogenetic trees constructed with the sequences of Indian parasite isolates. **a** Phylogenetic tree constructed with the sequences of parasite isolates with the global reference panel. **b** Phylogenetic tree constructed for internal comparison of the sequences of parasite isolates. Lb(P):*L. braziliensis* [Peru] (Skin); Ld(DD8)/Ld(IN): *L. donovani* [India] (Bone marrow); Li(SP):*L*. *infantum* [Spain] (Unknown); Lm(A1):*L. major* [Libya]; Lt(TK):*L. tropica* [Turkey] (Skin); Lam: *L*. *amazonenis* (Skin); Li(TK):*L*. *infantum* [Turkey] (Spleen), IN-VL; *L. donovani* [India] (Bone marrow), IN-PKDL; *Leishmania* from PKDL [India] (Skin)

**Fig. 5:**
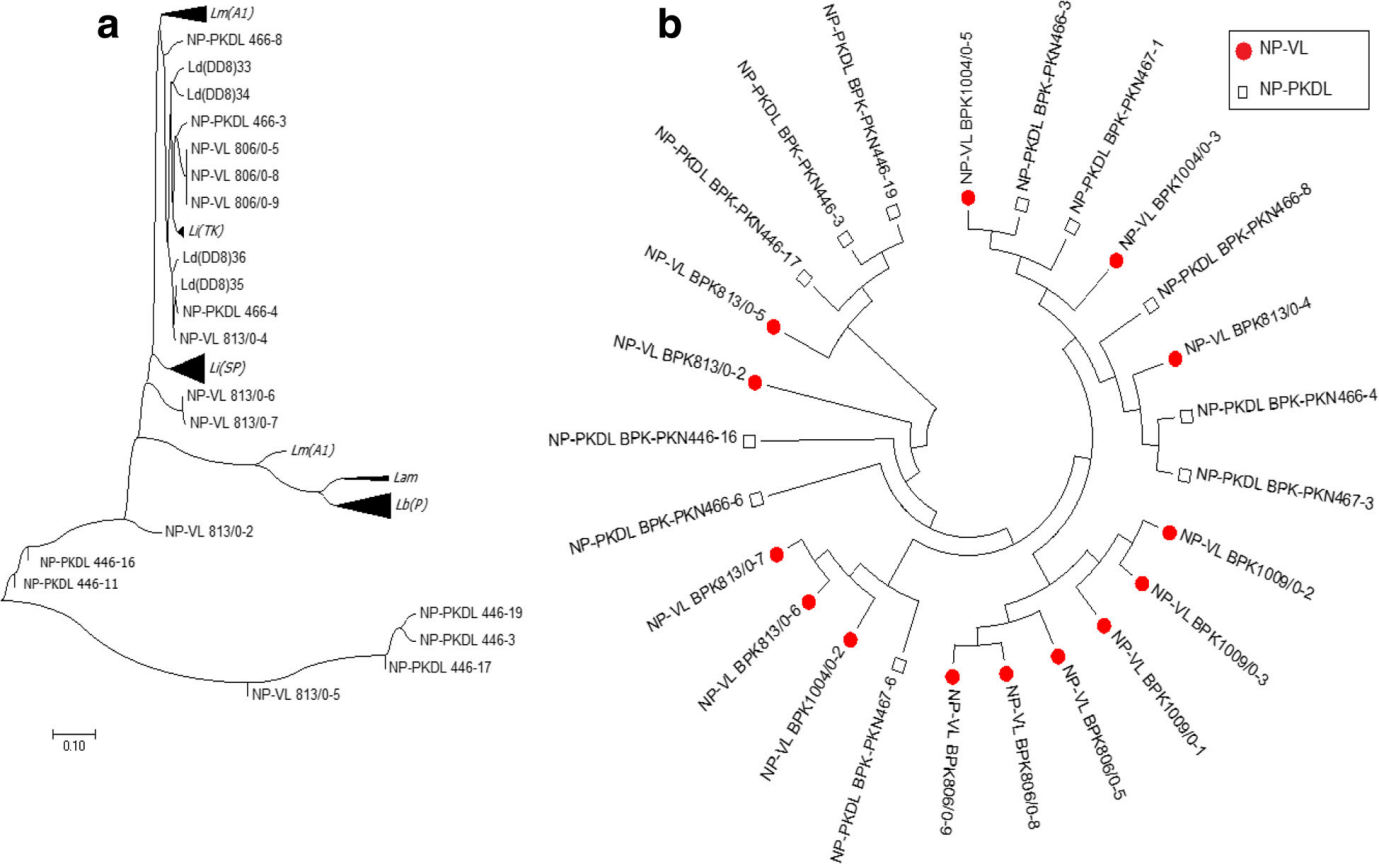
Phylogenetic trees constructed with the sequences of Nepal parasite isolates. **a** Phylogenetic tree constructed with the sequences of parasite isolates with the global reference panel. **b** Phylogenetic tree constructed for internal comparison of the sequences of parasite isolates. Lb(P): *L. braziliensis* [Peru] (Skin);Ld(DD8)/Ld(IN):*L. donovani* [India] (Bone marrow); Li(SP):*L. infantum* [Spain] (Unknown); Lm(A1):*L. major* [Libya]; Lt(TK):*L. tropica* [Turkey] (Skin); Lam:*L. amazonenis* (Skin); Li(TK):*L. infantum* [Turkey] (Spleen); NP-VL: Leishmania VL Nepalian isolate (Bone marrow); NP-PKDL: Leishmania cause PKDL [Nepal] (Skin)

Similar analyses were conducted for the isolates from India and Nepal. The sequences of the Indian and Nepalese parasites causing VL and post kala-azar dermal leishmaniasis (PKDL), clustered within the VL group of the reference panel positioned closer to *L. donovani and L. infantum* (Figs. 3a, 4a and 5a), while CL causing parasites obtained from skin lesions of Indian patients, formed a sister cluster with *L. tropica* (Fig. 4). However, there were no distinct subgroups within *L. donovani* that induce either VL or PKDL either in India or Nepal (Fig. 3a). This lack of clustering was further confirmed via internal comparison of Indian and Nepalese sequences (Figs. 4 and 5). None of the Sri Lankan, Indian or Nepalese isolates clustered with *L. major*, *L. braziliensis* or *L. amazonensis* (Figs. 3a, 4a and 5a).

## Discussion

This study was aimed to examine genetic diversity, population structure, phylogenetic relationships as well as the association between genotypic and phenotypic characteristics of *L. donovani* in Sri Lanka with the use of a minicircle-based strain specific sequence diversity assay. Attempts were also made to investigate Sri Lankan *L. donovani* sequences in relation to those in the neighboring countries of India and Nepal as well as the rest of the world. Distinct genetic clustering of Sri Lankan parasites according to their phenotypic properties, coupled with an admixed nature of the population was observed for the first time in Sri Lanka.

In the multiple sequence alignment, unique conserved regions could be clearly identified with minor gaps and nucleotide mismatches, since such regions remain conserved across many species and between classes of the same species. The existence of minicircle sequence haplotype diversity among different species and within the same species of *Leishmania* is fairly well established [41, 57–61]. The haplotype diversity value based on minicircle sequences revealed the degree of diversity within the Sri Lankan isolates with different phenotypic properties. Furthermore, lack of clear genotypic pattern in relation to geography within the country was revealed, indicating a population mix or presence of gene flow between parasite populations that may be still in the process of expansion. High levels of haplotype and nucleotide diversity were observed in Sri Lankan, Nepalese and Indian sequences. However, the highest haplotype diversity and nucleotide diversity were observed for the Indian parasite sequences indicating the highly diverse nature of the Indian isolates when compared to the Sri Lankan and Nepalese counterparts.

Indeed, a recent population expansion or an existence of an excess number of alleles often observed in genetic hitch-hiking was evident from Fu’s *F*s test for Sri Lankan sequences, where a negative Tajima’s D test implies purifying selection. In contrast, positive *F*s value observed in both Nepal and Indian sequences are an indication of deficient alleles, suggestive of a recent population bottleneck, and a positive Tajima’s D points towards a balancing selection.

The degree of variability within *Leishmania* species has been linked to the number of vectors and/or animal reservoir(s) involved in the transmission of these parasites [62, 63]. Therefore, the high variability found in *Leishmania* isolates in Sri Lanka, could be due to its transmission associated with several sub-populations of sand flies that belong to the genus *Phlebotomus* reported from various parts of Sri Lanka [64, 65].

This study confirms that, sequences of the Sri Lankan, Indian and Nepalese (VL and PKDL) parasites, clustered within the *L. donovani* complex, while CL causing parasites obtained from skin lesions of Indian patients, formed a sister cluster with *L. tropica.* None of the Sri Lankan CL and VL parasites were found to be associated with *L. infantum,* a closely related subspecies within the *L. donovani* complex [5, 61]. However, within the *L. donovani* group of sequences, the Sri Lankan parasites formed a distinct cluster, in agreement with previous observations made using different genotyping tools [8]. This may indicate the parasite’s existence over a prolonged period within the country, though the clinical cases were recognized only recently [11]. Interestingly, the Sri Lankan parasites belonged to a group, which was well differentiated from other dermotropic *Leishmania* parasites viz.*, L. tropica, L. major, L. amazonensis and L. braziliensis* indicating potential population isolation*.*

There were three distinct sub-clusters seen within the Sri Lankan group, comprising sequences from visceral leishmaniasis (VL) patients, cutaneous leishmaniasis patients (CL-S), and CL cases that showed poor response to the standard drug SSG (CL-DR). However, no specific clustering was observed in relation to their geographical origins within Sri Lanka. The phylogenetic differentiation within CL-S, CL-DR and VL may be due to the genetic mutations/variations associated with these three clinical groups (viz. VL, uncomplicated CL and CL with poor response to antimonial drugs, which is currently used as the first line of treatment in Sri Lanka) [66].

It is possible that multiple genetic strains of *L. donovani* exist within Sri Lanka*,* as reported from other geographic regions in the world [19, 31, 35]. Occasional reports on atypical cutaneous leishmaniasis reported in literature, indicate that more than one strain of *L. donovani* is likely to cause cutaneous disease [5, 6, 29]. *L. donovani* strains of the same genetic and/or geographical group are also known to result in different clinical outcomes [35]. This study with Sri Lankan isolates further affirms that there is sub-genetic diversity in *L. donovani* in the Indian subcontinent, which warrants further investigation on how that diversity translates into pathogenesis and its implications on patient management and treatment outcome.

Sri Lankan *L. donovani* parasites are likely to have evolved more recently as a novel group compared to other members of the genus, since the sequences from these isolates were found in the most peripheral branches of the phylogram. Given Sri Lanka’s geographic position as an island close to India, the parasites may have been in circulation within the country long enough to adopt such independent genetic characteristics. However, it is unlikely that these parasites were introduced within the last 2–3 decades, when the disease became apparent. As suggested by past genetic studies, the *L. donovani* complex has originated 3–4 million years ago [31, 35]. Initial cases reported in Sri Lanka about 30 years ago were all imported cases from the Middle East and Africa [67, 68]. *Leishmania* parasites are known to find new ecologies along with the immigration of people [55, 68, 69]. It is possible that the parasites may have been introduced to Sri Lanka by locals returning after employment overseas or by foreign immigrants [5, 68]. Sri Lankan *Leishmania* parasites may have co-evolved with the ecological settings along with its vector and possible reservoir hosts. However, this remains mere speculation with the precise origin of the Sri Lankan *L. donovani* parasites being debatable and not addressed through the present study.

Use of the kDNA minicircle region can be used as a surrogate genotyping tool for the identification of species-specific sequence footprints among different species of *Leishmania* and for the identification of new *Leishmania* species or strains with relative ease and reasonable accuracy. Such tools can be cost-effective in resource limited settings and provide for rapid identification of parasite species. However, such data obtained are unlikely to provide accurate information on evolutionary history of the parasite, which in turn may require high resolution evaluation of the whole genome and sequence analysis of markers that are representative of the entire parasite genome.

## Conclusion

For the first time this study reveals the extent of genetic diversity of *L. donovani* in Sri Lanka and a clear clustering nature of local parasites according to their antimonial sensitivity and tissue localization. Furthermore, the genetic differentiation between CL-DR and CL-S suggests a likely genetic basis for poor responsiveness to antimonial drugs and possible drug resistance. Overall, genetic variations associated with specific functional characteristics are likely to influence the disease phenotype, which is of clinical relevance and significance. Studies are underway to use whole genome sequence information with more detailed and higher resolution mapping, to investigate the parasite genetic variations associated with distinct clinical manifestations.

## Additional files

Additional file 1: Multiple sequence alignment. Multiple sequence alignment of the Sri Lankan, Indian and Nepalese isolates along with the reference sequences using CLUSTAL 2.1 multiple sequence alignment. Dashes(−) represent gaps introduced to optimize alignment. Asteriks (*) represent consensus nucleotides in the sequence. Lb(P):*L. braziliensis* [Peru] (Skin);Ld(DD8)/Ld(IN):*L. donovani* [India] (Bone marrow); Li(SP):*L. infantum* [Spain] (Unknown); Lm(A1):*L. major* [Libya]; Lt(TK):*L. tropica* [Turkey] (Skin); Lam: *L. amazonenis* (Skin); Li(TK):*L. infantum* [Turkey] (Spleen); SL-VL (52–55): VL Sri Lankan isolate (Bone marrow); SL_CL-S:*Leishmania* CL Sri Lankan isolate (Skin); SL_CL-DR:*Leishmania* CL Sri Lankan that failed to respond to antimonial drugs (Skin); IN-VL: *L. donovani* [India] (Bone marrow); IN-PKDL: *Leishmania* from post-kala azar dermal leishmaniasis (PKDL) [India] (Skin); NP-VL: *Leishmania* VL Nepalian isolate (Bone marrow); NP-PKDL:*Leishmania* cause PKDL [Nepal] (Skin). (DOCX 25 kb)

Additional file 2: The isolation by distance analysis showed the linear correlation between genetic diversity and geographic distance. Three populations were defined such as Sri Lanka, India and Nepal. However, this correlation was not statistically significant. (DOC 175 kb)

## Abbreviations

CL: Cutaneous leishmaniasis
FBS: Fetal bovine serum
FST: Pair wise fixation index
gp63: Glycoprotein 63
Hd: Haplotype diversity
IL-SSG: Intra-lesional sodium stibogluconate
ITS: Internal transcribed spacer
kDNA: Kinetoplastid DNA
LPG: Lipophosphoglycan
MCL: Muco-cutaneous leishmaniasis
MSA: Multiple sequence alignment
Nd: Nucleotide diversity
PCR: Polymerase Chain Reaction
PKDL: Post kala-azar dermal leishmaniasis
SL-CL-DR: Cutaneous leishmaniasis that showed poor response to antimony therapy in Sri Lanka
SL-CL-S: Uncomplicated cutaneous leishmaniasis in Sri Lanka
SL-VL: Visceral disease in Sri Lanka
VL: Visceral leishmaniasis
