# Supplementary material for: Genetic diversity of Leishmania donovani that causes cutaneous leishmaniasis in Sri Lanka: a cross sectional study with regional comparisons
Source: BMC Infect Dis. 2017 Dec 22;17:791. doi: 10.1186/s12879-017-2883-x (PMC5741890; doi:10.1186/s12879-017-2883-x)
Supplement: Supplementary file 1 — Multiple sequence alignment. Multiple sequence alignment of the Sri Lankan, Indian and Nepalese isolates along with the reference sequences using CLUSTAL 2.1 multiple sequence alignment. Dashes(−) represent gaps introduced to optimize alignment. Asteriks (*) represent consensus nucleotides in the sequence. Lb(P):L. braziliensis [Peru] (Skin);Ld(DD8)/Ld(IN):L. donovani [India] (Bone marrow); Li(SP):L. infantum [Spain] (Unknown); Lm(A1):L. major [Libya]; Lt(TK):L. tropica [Turkey] (Skin); Lam: L. amazonenis (Skin); Li(TK):L. infantum [Turkey] (Spleen); SL-VL (52–55): VL Sri Lankan isolate (Bone marrow); SL_CL-S:Leishmania CL Sri Lankan isolate (Skin); SL_CL-DR:Leishmania CL Sri Lankan that failed to respond to antimonial drugs (Skin); IN-VL: L. donovani [India] (Bone marrow); IN-PKDL: Leishmania from post-kala azar dermal leishmaniasis (PKDL) [India] (Skin); NP-VL: Leishmania VL Nepalian isolate (Bone marrow); NP-PKDL:Leishmania cause PKDL [Nepal] (Skin). (DOCX 25 kb) [file 12879_2017_2883_MOESM1_ESM.docx]

**Additional files**

SL-CL-S_2-1 GGGTAGGGGCGTTCTGCGAAAAT-GG-AAAAATGG-CTGCAGAAATCCCG

SL-CL-S_2-6 GGGTAGGGGCGTTCTGCGAAAAT-GG-AAAAATGG-CTGCAGAAATCCCG

SL-CL-S_2-7 GGGTAGGGGCGTTCTGCGAAAAT-GG-AAAAATGG-CTGCAGAAATCCCG

SL-CL-S_2-8 GGGTAGGGGCGTTCTGCGAAAAT-GG-AAAAATGG-CTGCAGAAATCCCG

SL-CL-S_2-9 GGGTAGGGGCGTTCTGCGAAAAT-GG-AAAAATGG-CTGCAGAAATCCCG

SL-CL-S_2-10 GGGTAGGGGCGTTCTGCGAAAAT-GG-AAAAATGG-CTGCAGAAATCCCG

SL-CL-S_3-2 GGGTAGGGGCGTTCTGCGAAAAT-CG-AAAAATGC-GTGCAGAAACCCGG

SL-CL-S_3-4 GGGTAGGGGCGTTCTGCGAAAAT-CG-AAAAATGC-GTGCAGAAACCCGG

SL-CL-S_3-5 GGGTAGGGGCGTTCTGCGAAAAT-CG-AAAAATGC-GTGCAGAAACCCGG

SL-CL-S_3-7 GGGTAGGGGCGTTCTGCGAAAAT-CG-AAAAATGC-GTGCAGAAACCCGG

SL-CL-S_3-8 GGGTAGGGGCGTTCTGCGAAAAT-CG-AAAAATGC-GTGCAGAAACCCGG

SL-CL-S_3-9 GGGTAGGGGCGTTCTGCGAAAAT-CG-AAAAATGC-GTGCAGAAACCCGG

SL-CL-S_6-3 GGGTAGGGGCGTTCTGCGAAAAT-GG-AAAAATGG-GTGCACAAATCCGG

SL-CL-S_6-5 GGGTAGGGGCGTTCTGCGAAAAT-GG-AAAAATGG-GTGCACAAATCCGG

SL-CL-S_6-6 GGGTAGGGGCGTTCTGCGAAAAT-GG-AAAAATGG-GTGCACAAATCCGG

SL-CL-S_6-2 GGGTAGGGGCGTTCTGCGAAAAT-GG-AAAAATGG-GTGCACAAATCCGG

SL-CL-S_7-2 GGGTAGGGGCGTTCTGCGAAAAT-GG-AAAAATGG-GTGCAGAAATCCCG

SL-CL-S_7-6 GGGTAGGGGCGTTCTGCGAAAAT-GG-AAAAATGG-GTGCAGAAATCCCG

SL-CL-S_7-7 GGGTAGGGGCGTTCTGCGAAAAT-GG-AAAAATGG-GTGCAGAAATCCCG

SL-CL-S_7-8 GGGTAGGGGCGTTCTGCGAAAAT-GG-AAAAATGG-GTGCAGAAATCCCG

SL-CL-S_8-3 GGGTAGGGGCGTTCTGCGAAAAT-GG-AAAAATGG-GTGCAGAAATCCCG

SL-CL-S_8-4 GGGTAGGGGCGTTCTGCGAAAAT-GG-AAAAATGG-GTGCAGAAATCCCG

SL-CL-S_8-5 GGGTAGGGGCGTTCTGCGAAAAT-GG-AAAAATGG-GTGCAGAAATCCCG

SL-CL-S_8-6 GGGTAGGGGCGTTCTGCGAAAAT-GG-AAAAATGG-GTGCAGAAATCCCG

SL-CL-S_10-1 GGGTAGGGGCGTTCTGCGAAAAT-GG-AAAAATGG-GTGCAGAAATGGCG

SL-CL-S_10-2 GGGTAGGGGCGTTCTGCGAAAAT-GG-AAAAATGG-GTGCAGAAATGGCG

SL-CL-S_10-3 GGGTAGGGGCGTTCTGCGAAAAT-GG-AAAAATGG-GTGCAGAAATGGCG

SL-CL-S_12-1 GGGTAGGGGCGTTCTGCGAAAAT-CG-AAAAATGG-GTGCAGAAATGCCG

SL-CL-S_12-2 GGGTAGGGGCGTTCTGCGAAAAT-CG-AAAAATGG-GTGCAGAAATGCCG

SL-CL-S_12-3 GGGTAGGGGCGTTCTGCGAAAAT-CG-AAAAATGG-GTGCAGAAATGCCG

SL-CL-S_13-1 GGGTAGGGGCGTTCTGCGAAAAT-CG-AAAAATGG-GTGCAGAAATCGCG

SL-CL-S_13-2 GGGTAGGGGCGTTCTGCGAAAAT-CG-AAAAATGG-GTGCAGAAATCGCG

SL-CL-S_13-3 GGGTAGGGGCGTTCTGCGAAAAT-CG-AAAAATGG-GTGCAGAAATCGCG

SL-CL-S_14-1 GGGTAGGGGCGTTCTGCGAAAAT-GG-AAAAATGG-GTGCAGAAATGGGG

SL-CL-S_14-2 GGGTAGGGGCGTTCTGCGAAAAT-GG-AAAAATGG-GTGCAGAAATGGGG

SL-CL-S_14-3 GGGTAGGGGCGTTCTGCGAAAAT-GG-AAAAATGG-GTGCAGAAATGGGG

SL-CL-S_16-3 GGGTAGGGGCGTTCTGCGAAAAT-GG-AAAAATGG-GTGCAGAAATCCCG

SL-CL-S_16-4 GGGTAGGGGCGTTCTGCGAAAAT-GG-AAAAATGG-GTGCAGAAATCCCG

SL-CL-S_16-6 GGGTAGGGGCGTTCTGCGAAAAT-GG-AAAAATGG-GTGCAGAAATCCCG

SL-CL-S_31-1 GGGTAGGGGCGTTCTGCGAAAAT-GG-AAAAATGG-GTGCAGAAATCCCG

SL-CL-S_31-2 GGGTAGGGGCGTTCTGCGAAAAT-GG-AAAAATGG-GTGCAGAAATCCCG

SL-CL-S_31-3 GGGTAGGGGCGTTCTGCGAAAAT-GG-AAAAATGG-GTGCAGAAATCCCG

SL-CL-S_31-4 GGGTAGGGGCGTTCTGCGAAAAT-GG-AAAAATGG-GTGCAGAAATCCCG

SL-CL-S_31-5 GGGTAGGGGCGTTCTGCGAAAAT-GG-AAAAATGG-GTGCAGAAATCCCG

SL-CL-S_32-1 GGGTAGGGGCGTTCTGCGAAAAT-GG-AAAAATGG-GTGCAGAAATCCCC

SL-CL-S_32-2 GGGTAGGGGCGTTCTGCGAAAAT-GG-AAAAATGG-GTGCAGAAATCCCC

SL-CL-S_32-3 GGGTAGGGGCGTTCTGCGAAAAT-GG-AAAAATGG-GTGCAGAAATCCCC

SL-CL-S_32-4 GGGTAGGGGCGTTCTGCGAAAAT-GG-AAAAATGG-GTGCAGAAATCCCC

SL-CL-S_32-5 GGGTAGGGGCGTTCTGCGAAAAT-GG-AAAAATGG-GTGCAGAAATCCCC

SL-CL-S_33-1 GGGTAGGGGCGTTCTGCGAAAAT-GG-AAAAATGG-GTGCAGAAATCCCG

SL-CL-S_33-2 GGGTAGGGGCGTTCTGCGAAAAT-GG-AAAAATGG-GTGCAGAAATCCCG

SL-CL-S_33-3 GGGTAGGGGCGTTCTGCGAAAAT-GG-AAAAATGG-GTGCAGAAATCCCG

SL-CL-S_33-4 GGGTAGGGGCGTTCTGCGAAAAT-GG-AAAAATGG-GTGCAGAAATCCCG

SL-CL-S_33-5 GGGTAGGGGCGTTCTGCGAAAAT-GG-AAAAATGG-GTGCAGAAATCCCG

SL-CL-S_34-1 GGGTAGGGGCGTTCTGCGAAAAT-GG-AAAAATGG-GTGCAGAAATCCGC

SL-CL-S_34-2 GGGTAGGGGCGTTCTGCGAAAAT-GG-AAAAATGG-GTGCAGAAATCCGC

SL-CL-S_34-3 GGGTAGGGGCGTTCTGCGAAAAT-GG-AAAAATGG-GTGCAGAAATCCGC

SL-CL-S_34-4 GGGTAGGGGCGTTCTGCGAAAAT-GG-AAAAATGG-GTGCAGAAATCCGC

SL-CL-S_34-5 GGGTAGGGGCGTTCTGCGAAAAT-GG-AAAAATGG-GTGCAGAAATCCGC

SL-CL-S_35-1 GGGTAGGGGCGTTCTGCGAAAAT-GG-AAAAATGG-GTGCAGAAATGGCG

SL-CL-S_35-3 GGGTAGGGGCGTTCTGCGAAAAT-GG-AAAAATGG-GTGCAGAAATGGCG

SL-CL-S_35-4 GGGTAGGGGCGTTCTGCGAAAAT-GG-AAAAATGG-GTGCAGAAATGGCG

SL-CL-S_35-6 GGGTAGGGGCGTTCTGCGAAAAT-GG-AAAAATGG-GTGCAGAAATGGCG

SL-CL-S_36-1 GGGTAGGGGCGTTCTGCGAAAAT-GG-AAAAATGG-GTGGAGAAATCCCG

SL-CL-S_36-2 GGGTAGGGGCGTTCTGCGAAAAT-GG-AAAAATGG-GTGGAGAAATCCCG

SL-CL-S_36-3 GGGTAGGGGCGTTCTGCGAAAAT-GG-AAAAATGG-GTGGAGAAATCCCG

SL-CL-S_36-4 GGGTAGGGGCGTTCTGCGAAAAT-GG-AAAAATGG-GTGGAGAAATCCCG

SL-CL-S_37-1 GGGTAGGGGCGTTCTGCGAAAAT-GG-AAAAATGG-GTCCAGAAATCCCG

SL-CL-S_37-2 GGGTAGGGGCGTTCTGCGAAAAT-GG-AAAAATGG-GTCCAGAAATCCCG

SL-CL-S_37-3 GGGTAGGGGCGTTCTGCGAAAAT-GG-AAAAATGG-GTCCAGAAATCCCG

SL-CL-S_37-4 GGGTAGGGGCGTTCTGCGAAAAT-GG-AAAAATGG-GTCCAGAAATCCCG

SL-CL-S_38-1 GGGTAGGGGCGTTCTGCGAAAAT-GG-AAAAATGG-GTGCAGAAATCCCG

SL-CL-S_38-2 GGGTAGGGGCGTTCTGCGAAAAT-GG-AAAAATGG-GTGCAGAAATCCCG

SL-CL-S_38-3 GGGTAGGGGCGTTCTGCGAAAAT-GG-AAAAATGG-GTGCAGAAATCCCG

SL-CL-S_38-4 GGGTAGGGGCGTTCTGCGAAAAT-GG-AAAAATGG-GTGCAGAAATCCCG

SL-CL-S_38-5 GGGTAGGGGCGTTCTGCGAAAAT-GG-AAAAATGG-GTGCAGAAATCCCG

SL-CL-S_39-1 GGGTAGGGGCGTTCTGCGAAAAT-GG-AAAAATGG-GTGCAGAAATCCCG

SL-CL-S_39-2 GGGTAGGGGCGTTCTGCGAAAAT-GG-AAAAATGG-GTGCAGAAATCCCG

SL-CL-S_39-3 GGGTAGGGGCGTTCTGCGAAAAT-GG-AAAAATGG-GTGCAGAAATCCCG

SL-CL-S_39-4 GGGTAGGGGCGTTCTGCGAAAAT-GG-AAAAATGG-GTGCAGAAATCCCG

SL-CL-S_39-5 GGGTAGGGGCGTTCTGCGAAAAT-GG-AAAAATGG-GTGCAGAAATCCCG

SL-CL-S_40-1 GGGTAGGGGCGTTCTGCGAAAAT-GG-AAAAATGG-GTGCAGAAATCCCG

SL-CL-S_40-3 GGGTAGGGGCGTTCTGCGAAAAT-GG-AAAAATGG-GTGCAGAAATCCCG

SL-CL-S_40-4 GGGTAGGGGCGTTCTGCGAAAAT-GG-AAAAATGG-GTGCAGAAATCCCG

SL-CL-S_40-5 GGGTAGGGGCGTTCTGCGAAAAT-GG-AAAAATGG-GTGCAGAAATCCCG

SL-CL-S_41-1 GGGTAGGGGCGTTCTGCGAAAAT-GG-AAAAATGG-GTGCACAAATCCCG

SL-CL-S_41-2 GGGTAGGGGCGTTCTGCGAAAAT-GG-AAAAATGG-GTGCACAAATCCCG

SL-CL-S_41-4 GGGTAGGGGCGTTCTGCGAAAAT-GG-AAAAATGG-GTGCACAAATCCCG

SL-CL-S_41-5 GGGTAGGGGCGTTCTGCGAAAAT-GG-AAAAATGG-GTGCACAAATCCCG

SL-CL-S_42-2 GGGTAGGGGCGTTCTGCGAAAAT-GG-AAAAATGG-GTGCAGAAATCCGG

SL-CL-S_42-3 GGGTAGGGGCGTTCTGCGAAAAT-GG-AAAAATGG-GTGCAGAAATCCGG

SL-CL-S_42-5 GGGTAGGGGCGTTCTGCGAAAAT-GG-AAAAATGG-GTGCAGAAATCCGG

SL-CL-S_43-1 GGGTAGGGGCGTTCTGCGAAAAT-GG-AAAAATGG-GTCCAGAAATCCCG

SL-CL-S_43-3 GGGTAGGGGCGTTCTGCGAAAAT-GG-AAAAATGG-GTCCAGAAATCCCG

SL-CL-S_43-4 GGGTAGGGGCGTTCTGCGAAAAT-GG-AAAAATGG-GTCCAGAAATCCCG

SL-CL-S_43-5 GGGTAGGGGCGTTCTGCGAAAAT-GG-AAAAATGG-GTCCAGAAATCCCG

SL-CL-S_43-6 GGGTAGGGGCGTTCTGCGAAAAT-GG-AAAAATGG-GTCCAGAAATCCCG

SL-CL-S_44-1 GGGTAGGGGCGTTCTGCGAAAAT-GG-AAAAATGG-GTGCACAAATCCCG

SL-CL-S_44-2 GGGTAGGGGCGTTCTGCGAAAAT-GG-AAAAATGG-GTGCACAAATCCCG

SL-CL-S_44-3 GGGTAGGGGCGTTCTGCGAAAAT-GG-AAAAATGG-GTGCACAAATCCCG

SL-CL-S_44-6 GGGTAGGGGCGTTCTGCGAAAAT-GG-AAAAATGG-GTGCACAAATCCCG

SL-CL-S_45-3 GGGTAGGGGCGTTCTGCGAAAAT-GG-AAAAATGG-GAGCAGAAATCCCG

SL-CL-S_46-1 GGGTAGGGGCGTTCTGCGAAAAT-GG-AAAAATGG-GTCGAGAAATCCCG

SL-CL-S_46-2 GGGTAGGGGCGTTCTGCGAAAAT-GG-AAAAATGG-GTCGAGAAATCCCG

SL-CL-S_46-4 GGGTAGGGGCGTTCTGCGAAAAT-GG-AAAAATGG-GTCGAGAAATCCCG

SL-CL-S_46-6 GGGTAGGGGCGTTCTGCGAAAAT-GG-AAAAATGG-GTCGAGAAATCCCG

SL-CL-S_48-1 GGGTAGGGGCGTTCTGCGAAAAT-GG-AAAAATGG-GTGCAGAAATCCGC

SL-CL-S_48-3 GGGTAGGGGCGTTCTGCGAAAAT-GG-AAAAATGG-GTGCAGAAATCCGC

SL-CL-S_49-4 GGGTAGGGGCGTTCTGCGAAAAT-GG-AAAAATGG-GTGCACAAATCCCG

SL-CL-S_49-5 GGGTAGGGGCGTTCTGCGAAAAT-GG-AAAAATGG-GTGCACAAATCCCG

SL-CL-S_50-1 GGGTAGGGGCGTTCTGCGAAAAT-GC-AAAAATGC-GTGCAGAAATCCCG

SL-CL-S_50-4 GGGTAGGGGCGTTCTGCGAAAAT-GC-AAAAATGC-GTGCAGAAATCCCG

SL-CL-S_50-6 GGGTAGGGGCGTTCTGCGAAAAT-GC-AAAAATGC-GTGCAGAAATCCCG

SL-CL-DR_1-2 GGGTAGGGGCGTTCTGCGAAAAT-GG-AAAAATGG-GTCGAGAAATCCCG

SL-CL-DR_1-4 GGGTAGGGGCGTTCTGCGAAAAT-GG-AAAAATGG-GTCGAGAAATCCCG

SL-CL-DR_1-6 GGGTAGGGGCGTTCTGCGAAAAT-GG-AAAAATGG-GTCGAGAAATCCCG

SL-CL-DR_1-7 GGGTAGGGGCGTTCTGCGAAAAT-GG-AAAAATGG-GTCGAGAAATCCCG

SL-CL-DR_1-8 GGGTAGGGGCGTTCTGCGAAAAT-GG-AAAAATGG-GTCGAGAAATCCCG

SL-CL-DR_1-9 GGGTAGGGGCGTTCTGCGAAAAT-GG-AAAAATGG-GTCGAGAAATCCCG

SL-CL-DR_1-10 GGGTAGGGGCGTTCTGCGAAAAT-GG-AAAAATGG-GTCGAGAAATCCCG

SL-CL-DR_9-1 GGGTAGGGGCGTTCTGCGAAAAT-GG-AAAAATGG-GTGCAGAAATCCCG

SL-CL-DR_9-2 GGGTAGGGGCGTTCTGCGAAAAT-GG-AAAAATGG-GTGCAGAAATCCCG

SL-CL-DR_9-3 GGGTAGGGGCGTTCTGCGAAAAT-GG-AAAAATGG-GTGCAGAAATCCCG

SL-CL-DR_9-4 GGGTAGGGGCGTTCTGCGAAAAT-GG-AAAAATGG-GTGCAGAAATCCCG

SL-CL-DR_11-1 GGGTAGGGGCGTTCTGCGAAAAT-GG-AAAAATGG-GTGCAGAAATCCCG

SL-CL-DR_11-4 GGGTAGGGGCGTTCTGCGAAAAT-GG-AAAAATGG-GTGCAGAAATCCCG

SL-CL-DR_15-1 GGGTAGGGGCGTTCTGCGAAAAT-GG-AAAAATGC-GTCCAGAAATCCCG

SL-CL-DR_15-5 GGGTAGGGGCGTTCTGCGAAAAT-GG-AAAAATGC-GTCCAGAAATCCCG

SL-CL-DR_51-2 GGGTAGGGGCGTTCTGCGAAAAT-GG-AAAAATGG-GTGCAGAAATCCCG

SL-CL-DR_51-3 GGGTAGGGGCGTTCTGCGAAAAT-GG-AAAAATGG-GTGCAGAAATCCCG

SL-CL-DR_51-4 GGGTAGGGGCGTTCTGCGAAAAT-GG-AAAAATGG-GTGCAGAAATCCCG

SL-CL-DR_51-5 GGGTAGGGGCGTTCTGCGAAAAT-GG-AAAAATGG-GTGCAGAAATCCCG

SL-CL-DR_56-1 GGGTAGGGGCGTTCTGCGAAAAT-GC-AAAAATCG-CTGCAGAAATCCCG

SL-CL-DR_56-2 GGGTAGGGGCGTTCTGCGAAAAT-GC-AAAAATCG-CTGCAGAAATCCCG

SL-CL-DR_56-4 GGGTAGGGGCGTTCTGCGAAAAT-GC-AAAAATCG-CTGCAGAAATCCCG

SL-CL-DR_56-5 GGGTAGGGGCGTTCTGCGAAAAT-GC-AAAAATCG-CTGCAGAAATCCCG

SL-VL_52-1 GGGTAGGGGCGTTCTGCGAAAAT-GG-AAAAATGG-GTGCAGAAATCCCG

SL-VL_52-2 GGGTAGGGGCGTTCTGCGAAAAT-GG-AAAAATGG-GTGCAGAAATCCCG

SL-VL_52-3 GGGTAGGGGCGTTCTGCGAAAAT-GG-AAAAATGG-GTGCAGAAATCCCG

SL-VL_52-4 GGGTAGGGGCGTTCTGCGAAAAT-GG-AAAAATGG-GTGCAGAAATCCCG

SL-VL_52-5 GGGTAGGGGCGTTCTGCGAAAAT-GG-AAAAATGG-GTGCAGAAATCCCG

SL-VL_52-6 GGGTAGGGGCGTTCTGCGAAAAT-GG-AAAAATGG-GTGCAGAAATCCCG

SL-VL_53-1 GGGTAGGGGCGTTCTGCGAAAAT-GG-AAAAATGG-GTGCAGAAATCCCG

SL-VL_53-2 GGGTAGGGGCGTTCTGCGAAAAT-GG-AAAAATGG-GTGCAGAAATCCCG

SL-VL_53-3 GGGTAGGGGCGTTCTGCGAAAAT-GG-AAAAATGG-GTGCAGAAATCCCG

SL-VL_53-4 GGGTAGGGGCGTTCTGCGAAAAT-GG-AAAAATGG-GTGCAGAAATCCCG

SL-VL_53-5 GGGTAGGGGCGTTCTGCGAAAAT-GG-AAAAATGG-GTGCAGAAATCCCG

SL-VL_54-1 GGGTAGGGGCGTTCTGCGAAAAT-GG-AAAAATGG-GTGCAGAAATCGCG

SL-VL_54-2 GGGTAGGGGCGTTCTGCGAAAAT-GG-AAAAATGG-GTGCAGAAATCGCG

SL-VL_54-3 GGGTAGGGGCGTTCTGCGAAAAT-GG-AAAAATGG-GTGCAGAAATCGCG

SL-VL_54-4 GGGTAGGGGCGTTCTGCGAAAAT-GG-AAAAATGG-GTGCAGAAATCGCG

SL-VL_54-5 GGGTAGGGGCGTTCTGCGAAAAT-GG-AAAAATGG-GTGCAGAAATCGCG

SL-VL_55-1 GGGTAGGGGCGTTCTGCGAAAAT-GG-AAAAATGG-CTGCAGAAATCCCG

SL-VL_55-2 GGGTAGGGGCGTTCTGCGAAAAT-GG-AAAAATGG-CTGCAGAAATCCCG

SL-VL_55-4 GGGTAGGGGCGTTCTGCGAAAAT-GG-AAAAATGG-CTGCAGAAATCCCG

SL-VL_55-5 GGGTAGGGGCGTTCTGCGAAAAT-GG-AAAAATGG-CTGCAGAAATCCCG

SL-VL_55-6 GGGTAGGGGCGTTCTGCGAAAAT-GG-AAAAATGG-CTGCAGAAATCCCG

IN-CL_10-171 GGGTAGGGGCGTTCTGCGAAAAT-CG-AAAAATGG-GTGCAGAAAACCCG

IN-CL_10-170 GGGTAGGGGCGTTCTGCGAAAAT-CG-AAAAATGG-GTGCAGAAAACCCG

IN-CL_10-173 GGGTAGGGGCGTTCTGCGAAAAT-CG-AAAAATGG-GTGCAGAAAACCCG

IN-CL_10-1731 GGGTAGGGGCGTTCTGCGAAAAT-CG-AAAAATGG-GTGCAGAAAACCCG

IN-PKDL_83-166 GGGTAGGGGCGTTCTGCGAAATC-GG-AAAAATGG-GTGCAGAAATCCCG

IN-PKDL_83-165 GGGTAGGGGCGTTCTGCGAAAAT-GG-AAAAATGG-GTGCAGAAATCCCG

IN-PKDL_83-168 GGGTAGGGGCGTTCTGCGAAAAC-CG-AAAAATGG-GTGCAGAAATCCCG

IN-VL_220-41 GGGTAGGGGCGTTCTGCGAAAAC-CG-AAAAATGG-GTGCAGAAATCCCG

IN-VL_220-50 GGGTAGGGGCGTTCTGCGAAAAC-CG-AAAAATGG-GTGCAGAAATCCCG

IN-VL_220-51 GGGTAGGGGCGTTCTGCGAAAAC-CG-AAAAATGG-GTGCAGAAATCCCG

Ld_DD8_33 GGGTAGGGGCGTTCTGCGAAAAT-GG-AAAAATGG-GTGCAGAAATCCCG

Ld_DD8_34 GGGTAGGGGCGTTCTGCGAAAAT-GG-AAAAATGG-GTGCAGAAATCCCG

Ld_DD8_35 GGGTAGGGGCGTTCTGCGAAAAC-CG-AAAAATGG-GTGCAGAAATCCCG

Ld_DD8_36 GGGTAGGGGCGTTCTGCGAAAAT-GG-AAAAATGG-GTGCAGAAATCCCG

NP-PKDL_446-16 GGGTAGGGGCGTTCTGCGAAATC-GC-GAAAATCC-ATACAGAAACCCCG

NP-PKDL_466-3 GGGTAGGGGCGTTCTGCGAAATC-GC-GAAAATCC-ATACAGAAACCCCG

NP-PKDL_446-17 GGGTAGGGGCGTTCTGCGAAATC-GC-GAAAATCC-ATACAGAAACCCCG

NP-PKDL_446-19 GGGTAGGGGCGTTCTGCGAAATC-GC-GAAAATCC-ATACAGAAACCCCG

NP-VL_813-6 GGGTAGGGGCGTTCTGCGAAAAC-CG-AAAAATGG-GTGCAGAAATCCCG

NP-VL_813-2 GGGTAGGGGCGTTCTGCGAAAAT-GG-AAAAATGG-GTGCAGAAATCCCG

NP-VL_813-7 GGGTAGGGGCGTTCTGCGAAAAC-CG-AAAAATGG-GTGCAGAAACCCCG

Lm_A1_18 GGGTAGGGGCGTTCTGCGAAAAC-CG-AAAAATGG-GTGCAGAAATCCCG

Lm_A1_16 GGGTAGGGGCGTTCTGCGAAATT-CG-AAAAATGG-GTGCAGAAATCCCG

Lm_A1_20 GGGTAGGGGCGTTCTGCGAAATT-CG-AAAAATGG-GTGCAGAAACCCCG

Lm_A1_10 GGGTAGGGGCGTTCTGCGAAAAC-TG-AAAAATGG-GTGCAGAAATCCCG

Lb_P_59 GGGTAGGGGCGTTCTGCGAAAACCTC-AAAAATGA-GTGCAGAAACCCCG

Lb_P_60 GGGTAGGGGCGTTCTGCGAAATTCGG-AAAAATGA-GTGCAGAAACCCCG

Lb_P_61 GGGTAGGGGCGTTCTGCGAAATCCTC-AAAAATGA-GTGCAGAAACCCCG

Lb_P_81 GGGTAGGGGCGTTCTGCGAAAAT-GGGGAAAATGA-GTGCAGAAACCCCG

Lb_P_80 GGGTAGGGGCGTTCTGCGAAATTCGG-AAAAATGA-GTGCAGAAACCCCG

Lb_P_62 GGGTAGGGGCGTTCTGCGAAATTCGG-AAAAATGA-GTGCAGAAACCCCG

Li-TK-89 GGGTAGGGGCGTTCTGCGAAATC-GG-GAAAATGG-GTGCAGAAATCCCG

Li-TK-85 GGGTAGGGGCGTTCTGCGAAATC-GG-AAAAATGG-GTGCAGAAATCCCG

Li-TK-83 GGGTAGGGGCGTTCTGCGAAATC-GG-AAAAAAGG-GTGCAGAAATCCCG

Li-TK-91 GGGTAGGGGCGTTCTGCGAAATC-GG-AAAAATGG-GTGCAGAAATCCCG

Li-TK-84 GGGTAGGGGCGTTCTGCGAAATC-GG-AAAAATGG-GTGCAGAAATCCCG

Li_SP_57 GGGTAGGGGCGTTCTGCGAAATC-GG-AAAAATGG-GTGCAGAAATCCCG

Li_SP_54 GGGTAGGGGCGTTCTGCGAAAAC-CG-AAAAATGG-GTGCAGAAATCCCG

Li_SP_52 GGGTAGGGGCGTTCTGCGAAAAC-CG-AAAAATGG-GTGCAGAAATCCCG

Li_SP_56 GGGTAGGGGCGTTCTGCGAAATC-GG-AAAAATGG-GTGCAGAAATCCCG

Li_SP_53 GGGTAGGGGCGTTCTGCGAAATC-GG-AAAAATGG-GTGCAGAAATCCCG

Li_SP_55 GGGTAGGGGCGTTCTGCGAAATC-GG-AAAAATGG-GTGCAGAAATCCCG

Lm_A1_9 GGGTAGGGGCGTTCTGCGAAAAC-CG-AAAAATGG-GTGCAGAAATCCCG

Lm_A1_7 GGGTAGGGGCGTTCTGCGAAAAC-CG-AAAAATGG-GTGCAGAAATCCCG

Lm_A1_17 GGGTAGGGGCGTTCTGCGAAAAC-TG-AAAAATGG-GTGCAGAAATCCCG

Lm_A1_19 GGGTAGGGGCGTTCTGCGAAAAT-GG-GAAAAGGGCATACAGAAACCCCG

Lt_TK_3 GGGTAGGGGCGTTCTGCGAAAATCGG-GAAAATTG-ATACAGAAACCCCG

Lt_TK_14 GGGTAGGGGCGTTCTGCGAAAAT-CG-AAAAATCG-ATACAGAAACCCCG

Lt_TK_5 GGGTAGGGGCGTTCTGCGAAAAT-CG-AAAAATCG-ATACAGAAACCCCG

Lam-322 GGGTAGGGGCGTTCTGCGAAAAT-GGCAAAAATGA-GTGCAGAAACCCCG

Lam-325 GGGTAGGGGCGTTCTGCGAAATC-GGCAAAAATGA-GTGCAGAAACCCCG

Lam-323 GGGTAGGGGCGTTCTGCGAAAACCGG-AAAAATGG-GTGCAGAAACCCCG

Lam-331 GGGTAGGGGCGTTCTGCGAAAAT-GGGAAAAATGA-GTGCAGAAACCCCG

********************* **** * ***

SL-CL-S_2-1 TTCAAAAA-TCGGC-GGAAAATGCGAAAA-ATCG-GCTCCGGGGCGG-GA

SL-CL-S_2-6 TTCAAAAA-TCGGC-GGAAAATGCGAAAA-ATCG-GCTCCGGGGCGG-GA

SL-CL-S_2-7 TTCAAAAA-TCGGC-GGAAAATGCGAAAA-ATCG-GCTCCGGGGCGG-GA

SL-CL-S_2-8 TTCAAAAA-TCGGC-GGAAAATGCGAAAA-ATCG-GCTCCGGGGCGG-GA

SL-CL-S_2-9 TTCAAAAA-TCGGC-GGAAAATGCGAAAA-ATCG-GCTCCGGGGCGG-GA

SL-CL-S_2-10 TTCAAAAA-TCGGC-GGAAAATGCGAAAA-ATCG-GCTCCGGGGCGG-GA

SL-CL-S_3-2 TTCAAAAA-TCGGC-GGAAAATGCCAAAA-ATCG-GGTCCGGGGCGG-GA

SL-CL-S_3-4 TTCAAAAA-TCGGC-GGAAAATGCCAAAA-ATCG-GGTCCGGGGCGG-GA

SL-CL-S_3-5 TTCAAAAA-TCGGC-GGAAAATGCCAAAA-ATCG-GGTCCGGGGCGG-GA

SL-CL-S_3-7 TTCAAAAA-TCGGC-GGAAAATGCCAAAA-ATCG-GGTCCGGGGCGG-GA

SL-CL-S_3-8 TTCAAAAA-TCGGC-GGAAAATGCCAAAA-ATCG-GGTCCGGGGCGG-GA

SL-CL-S_3-9 TTCAAAAA-TCGGC-GGAAAATGCCAAAA-ATCG-GGTCCGGGGCGG-GA

SL-CL-S_6-3 TTCAAAAA-TCGGC-GGAAAATGCCAAAA-ATCG-GCTCCGGGGCGG-GA

SL-CL-S_6-5 TTCAAAAA-TCGGC-GGAAAATGCCAAAA-ATCG-GCTCCGGGGCGG-GA

SL-CL-S_6-6 TTCAAAAA-TCGGC-GGAAAATGCCAAAA-ATCG-GCTCCGGGGCGG-GA

SL-CL-S_6-2 TTCAAAAA-TCGGC-GGAAAATGCCAAAA-ATCG-GCTCCGGGGCGG-GA

SL-CL-S_7-2 TTCAAAAA-TCGGC-GGAAAATGCCAAAA-ATCG-GCTCCGGGGCGG-GA

SL-CL-S_7-6 TTCAAAAA-TCGGC-GGAAAATGCCAAAA-ATCG-GCTCCGGGGCGG-GA

SL-CL-S_7-7 TTCAAAAA-TCGGC-GGAAAATGCCAAAA-ATCG-GCTCCGGGGCGG-GA

SL-CL-S_7-8 TTCAAAAA-TCGGC-GGAAAATGCCAAAA-ATCG-GCTCCGGGGCGG-GA

SL-CL-S_8-3 TTCAAAAA-TCGGC-GGAAAATGCCAAAA-ATCG-GCTCCGGGGCGG-GA

SL-CL-S_8-4 TTCAAAAA-TCGGC-GGAAAATGCCAAAA-ATCG-GCTCCGGGGCGG-GA

SL-CL-S_8-5 TTCAAAAA-TCGGC-GGAAAATGCCAAAA-ATCG-GCTCCGGGGCGG-GA

SL-CL-S_8-6 TTCAAAAA-TCGGC-GGAAAATGCCAAAA-ATCG-GCTCCGGGGCGG-GA

SL-CL-S_10-1 TTCAAAAA-TCGGC-GGAAAATGCCAAAA-ATCG-GCTCCGGGGCGG-GA

SL-CL-S_10-2 TTCAAAAA-TCGGC-GGAAAATGCCAAAA-ATCG-GCTCCGGGGCGG-GA

SL-CL-S_10-3 TTCAAAAA-TCGGC-GGAAAATGCCAAAA-ATCG-GCTCCGGGGCGG-GA

SL-CL-S_12-1 TTCAAAAA-TCGGC-GGAAAATGCCAAAA-ATCG-GCTCCGGGGCGG-GA

SL-CL-S_12-2 TTCAAAAA-TCGGC-GGAAAATGCCAAAA-ATCG-GCTCCGGGGCGG-GA

SL-CL-S_12-3 TTCAAAAA-TCGGC-GGAAAATGCCAAAA-ATCG-GCTCCGGGGCGG-GA

SL-CL-S_13-1 TTCAAAAA-TCGGC-GGAAAATGCCAAAA-ATCG-GCTCCGGGGCGG-GA

SL-CL-S_13-2 TTCAAAAA-TCGGC-GGAAAATGCCAAAA-ATCG-GCTCCGGGGCGG-GA

SL-CL-S_13-3 TTCAAAAA-TCGGC-GGAAAATGCCAAAA-ATCG-GCTCCGGGGCGG-GA

SL-CL-S_14-1 TTCAAAAA-TCGGC-GGAAAATGCCAAAA-ATCG-GCTCCGGGGCGG-GA

SL-CL-S_14-2 TTCAAAAA-TCGGC-GGAAAATGCCAAAA-ATCG-GCTCCGGGGCGG-GA

SL-CL-S_14-3 TTCAAAAA-TCGGC-GGAAAATGCCAAAA-ATCG-GCTCCGGGGCGG-GA

SL-CL-S_16-3 TTGAAAAA-TCGGC-GGAAATTGCCAAAA-ATCG-GCTCCGGGGCGG-GA

SL-CL-S_16-4 TTGAAAAA-TCGGC-GGAAATTGCCAAAA-ATCG-GCTCCGGGGCGG-GA

SL-CL-S_16-6 TTGAAAAA-TCGGC-GGAAATTGCCAAAA-ATCG-GCTCCGGGGCGG-GA

SL-CL-S_31-1 TTCAAAAA-TCGGC-GGAAAATGCCAAAA-ATCG-GCTCCGGGGCGG-GA

SL-CL-S_31-2 TTCAAAAA-TCGGC-GGAAAATGCCAAAA-ATCG-GCTCCGGGGCGG-GA

SL-CL-S_31-3 TTCAAAAA-TCGGC-GGAAAATGCCAAAA-ATCG-GCTCCGGGGCGG-GA

SL-CL-S_31-4 TTCAAAAA-TCGGC-GGAAAATGCCAAAA-ATCG-GCTCCGGGGCGG-GA

SL-CL-S_31-5 TTCAAAAA-TCGGC-GGAAAATGCCAAAA-ATCG-GCTCCGGGGCGG-GA

SL-CL-S_32-1 TTCAAAAA-TCGGC-GGAAAATGCCAAAA-ATCG-GCTCCGGGGCGG-GA

SL-CL-S_32-2 TTCAAAAA-TCGGC-GGAAAATGCCAAAA-ATCG-GCTCCGGGGCGG-GA

SL-CL-S_32-3 TTCAAAAA-TCGGC-GGAAAATGCCAAAA-ATCG-GCTCCGGGGCGG-GA

SL-CL-S_32-4 TTCAAAAA-TCGGC-GGAAAATGCCAAAA-ATCG-GCTCCGGGGCGG-GA

SL-CL-S_32-5 TTCAAAAA-TCGGC-GGAAAATGCCAAAA-ATCG-GCTCCGGGGCGG-GA

SL-CL-S_33-1 TACAAAAA-TCGGC-GGAAAATGCCAAAA-ATCG-GCTCCGGGGCGG-GA

SL-CL-S_33-2 TACAAAAA-TCGGC-GGAAAATGCCAAAA-ATCG-GCTCCGGGGCGG-GA

SL-CL-S_33-3 TACAAAAA-TCGGC-GGAAAATGCCAAAA-ATCG-GCTCCGGGGCGG-GA

SL-CL-S_33-4 TACAAAAA-TCGGC-GGAAAATGCCAAAA-ATCG-GCTCCGGGGCGG-GA

SL-CL-S_33-5 TACAAAAA-TCGGC-GGAAAATGCCAAAA-ATCG-GCTCCGGGGCGG-GA

SL-CL-S_34-1 TTCAAAAA-TGGGC-GGAAAATGCCAAAA-ATCG-GCTCGGGGGCGG-GA

SL-CL-S_34-2 TTCAAAAA-TGGGC-GGAAAATGCCAAAA-ATCG-GCTCGGGGGCGG-GA

SL-CL-S_34-3 TTCAAAAA-TGGGC-GGAAAATGCCAAAA-ATCG-GCTCGGGGGCGG-GA

SL-CL-S_34-4 TTCAAAAA-TGGGC-GGAAAATGCCAAAA-ATCG-GCTCGGGGGCGG-GA

SL-CL-S_34-5 TTCAAAAA-TGGGC-GGAAAATGCCAAAA-ATCG-GCTCGGGGGCGG-GA

SL-CL-S_35-1 TTCAAAAA-TCGGG-GGAAAATGCCAAAA-ATCG-GCTCCGGGGCGG-GA

SL-CL-S_35-3 TTCAAAAA-TCGGG-GGAAAATGCCAAAA-ATCG-GCTCCGGGGCGG-GA

SL-CL-S_35-4 TTCAAAAA-TCGGG-GGAAAATGCCAAAA-ATCG-GCTCCGGGGCGG-GA

SL-CL-S_35-6 TTCAAAAA-TCGGG-GGAAAATGCCAAAA-ATCG-GCTCCGGGGCGG-GA

SL-CL-S_36-1 TTGTAAAA-TCGGC-GGAAAATGCCAAAA-ATCG-GCTCCGGGGCGG-GA

SL-CL-S_36-2 TTGTAAAA-TCGGC-GGAAAATGCCAAAA-ATCG-GCTCCGGGGCGG-GA

SL-CL-S_36-3 TTGTAAAA-TCGGC-GGAAAATGCCAAAA-ATCG-GCTCCGGGGCGG-GA

SL-CL-S_36-4 TTGTAAAA-TCGGC-GGAAAATGCCAAAA-ATCG-GCTCCGGGGCGG-GA

SL-CL-S_37-1 TTCAAAAA-TCGGC-GGAAAATGCCAAAA-ATGG-GCTCCGGGGCGG-GA

SL-CL-S_37-2 TTCAAAAA-TCGGC-GGAAAATGCCAAAA-ATGG-GCTCCGGGGCGG-GA

SL-CL-S_37-3 TTCAAAAA-TCGGC-GGAAAATGCCAAAA-ATGG-GCTCCGGGGCGG-GA

SL-CL-S_37-4 TTCAAAAA-TCGGC-GGAAAATGCCAAAA-ATGG-GCTCCGGGGCGG-GA

SL-CL-S_38-1 TTCAAAAA-TCGGC-GGAAAATGGGAAAA-ATCG-GCTCCGGGGCGG-GA

SL-CL-S_38-2 TTCAAAAA-TCGGC-GGAAAATGGGAAAA-ATCG-GCTCCGGGGCGG-GA

SL-CL-S_38-3 TTCAAAAA-TCGGC-GGAAAATGGGAAAA-ATCG-GCTCCGGGGCGG-GA

SL-CL-S_38-4 TTCAAAAA-TCGGC-GGAAAATGGGAAAA-ATCG-GCTCCGGGGCGG-GA

SL-CL-S_38-5 TTCAAAAA-TCGGC-GGAAAATGGGAAAA-ATCG-GCTCCGGGGCGG-GA

SL-CL-S_39-1 TTCAAAAA-TCGGC-GAAAATCGCCAAAA-ATCG-GCTCCGGGGCGG-GA

SL-CL-S_39-2 TTCAAAAA-TCGGC-GAAAATCGCCAAAA-ATCG-GCTCCGGGGCGG-GA

SL-CL-S_39-3 TTCAAAAA-TCGGC-GAAAATCGCCAAAA-ATCG-GCTCCGGGGCGG-GA

SL-CL-S_39-4 TTCAAAAA-TCGGC-GAAAATCGCCAAAA-ATCG-GCTCCGGGGCGG-GA

SL-CL-S_39-5 TTCAAAAA-TCGGC-GAAAATCGCCAAAA-ATCG-GCTCCGGGGCGG-GA

SL-CL-S_40-1 TTCAAAAA-TCGGC-GCGAAATGCCAAAA-ATCG-GCTCCGGGGCGG-GA

SL-CL-S_40-3 TTCAAAAA-TCGGC-GCGAAATGCCAAAA-ATCG-GCTCCGGGGCGG-GA

SL-CL-S_40-4 TTCAAAAA-TCGGC-GCGAAATGCCAAAA-ATCG-GCTCCGGGGCGG-GA

SL-CL-S_40-5 TTCAAAAA-TCGGC-GCGAAATGCCAAAA-ATCG-GCTCCGGGGCGG-GA

SL-CL-S_41-1 TTCAAAAA-TCGGC-GGAAAATGCCAAAA-ATCG-GCTCGCGGGCGG-GA

SL-CL-S_41-2 TTCAAAAA-TCGGC-GGAAAATGCCAAAA-ATCG-GCTCGCGGGCGG-GA

SL-CL-S_41-4 TTCAAAAA-TCGGC-GGAAAATGCCAAAA-ATCG-GCTCGCGGGCGG-GA

SL-CL-S_41-5 TTCAAAAA-TCGGC-GGAAAATGCCAAAA-ATCG-GCTCGCGGGCGG-GA

SL-CL-S_42-2 TTCAAAAA-TCCGC-GGAAAATGGCAAAA-ATCG-GCTCCGGGGCGG-GA

SL-CL-S_42-3 TTCAAAAA-TCCGC-GGAAAATGGCAAAA-ATCG-GCTCCGGGGCGG-GA

SL-CL-S_42-5 TTCAAAAA-TCCGC-GGAAAATGGCAAAA-ATCG-GCTCCGGGGCGG-GA

SL-CL-S_43-1 TTCAAAAA-TCGGC-GGAAAATGCCAAAA-ATCG-GCTCCGGGGCGG-GA

SL-CL-S_43-3 TTCAAAAA-TCGGC-GGAAAATGCCAAAA-ATCG-GCTCCGGGGCGG-GA

SL-CL-S_43-4 TTCAAAAA-TCGGC-GGAAAATGCCAAAA-ATCG-GCTCCGGGGCGG-GA

SL-CL-S_43-5 TTCAAAAA-TCGGC-GGAAAATGCCAAAA-ATCG-GCTCCGGGGCGG-GA

SL-CL-S_43-6 TTCAAAAA-TCGGC-GGAAAATGCCAAAA-ATCG-GCTCCGGGGCGG-GA

SL-CL-S_44-1 TTCAAAAA-TCGGC-GCAAAATGCCAAAA-ATCG-GCTCCGGGGCGG-GA

SL-CL-S_44-2 TTCAAAAA-TCGGC-GCAAAATGCCAAAA-ATCG-GCTCCGGGGCGG-GA

SL-CL-S_44-3 TTCAAAAA-TCGGC-GCAAAATGCCAAAA-ATCG-GCTCCGGGGCGG-GA

SL-CL-S_44-6 TTCAAAAA-TCGGC-GCAAAATGCCAAAA-ATCG-GCTCCGGGGCGG-GA

SL-CL-S_45-3 TTCAAAAA-T-GCCGGGAAAATGCCAAAA-ATCG-GCTCCGGGGCGG-GA

SL-CL-S_46-1 TTCAAAAA-TCGGC-GGAAAATGCCAAAA-ATCG-GCTCCGGGGCGG-GA

SL-CL-S_46-2 TTCAAAAA-TCGGC-GGAAAATGCCAAAA-ATCG-GCTCCGGGGCGG-GA

SL-CL-S_46-4 TTCAAAAA-TCGGC-GGAAAATGCCAAAA-ATCG-GCTCCGGGGCGG-GA

SL-CL-S_46-6 TTCAAAAA-TCGGC-GGAAAATGCCAAAA-ATCG-GCTCCGGGGCGG-GA

SL-CL-S_48-1 TTCAAAAA-TCGGC-GCAAAATGCCAAAA-ATCG-GCTCCGGGGCGG-GA

SL-CL-S_48-3 TTCAAAAA-TCGGC-GCAAAATGCCAAAA-ATCG-GCTCCGGGGCGG-GA

SL-CL-S_49-4 TTCAAAAA-TCCCC-GGAAAATGCCAAAA-ATCG-GCTCCGGGGCGG-GA

SL-CL-S_49-5 TTCAAAAA-TCCCC-GGAAAATGCCAAAA-ATCG-GCTCCGGGGCGG-GA

SL-CL-S_50-1 TTCAAAAA-TCGGC-GGAAAATGCCAAAA-ATCG-GCTCCGGGGCGG-GA

SL-CL-S_50-4 TTCAAAAA-TCGGC-GGAAAATGCCAAAA-ATCG-GCTCCGGGGCGG-GA

SL-CL-S_50-6 TTCAAAAA-TCGGC-GGAAAATGCCAAAA-ATCG-GCTCCGGGGCGG-GA

SL-CL-DR_1-2 TTCAAAAA-TCGCT-CAAAAATGCCAAAA-ATG--CGTCCGGGGCGG-GA

SL-CL-DR_1-4 TTCAAAAA-TCGCT-CAAAAATGCCAAAA-ATG--CGTCCGGGGCGG-GA

SL-CL-DR_1-6 TTCAAAAA-TCGCT-CAAAAATGCCAAAA-ATG--CGTCCGGGGCGG-GA

SL-CL-DR_1-7 TTCAAAAA-TCGCT-CAAAAATGCCAAAA-ATG--CGTCCGGGGCGG-GA

SL-CL-DR_1-8 TTCAAAAA-TCGCT-CAAAAATGCCAAAA-ATG--CGTCCGGGGCGG-GA

SL-CL-DR_1-9 TTCAAAAA-TCGCT-CAAAAATGCCAAAA-ATG--CGTCCGGGGCGG-GA

SL-CL-DR_1-10 TTCAAAAA-TCGCT-CAAAAATGCCAAAA-ATG--CGTCCGGGGCGG-GA

SL-CL-DR_9-1 TTCAAAAA-TCGGT-CAAAAATGCCAAAA-ATG--GGTCCGGGGCGG-GA

SL-CL-DR_9-2 TTCAAAAA-TCGGT-CAAAAATGCCAAAA-ATG--GGTCCGGGGCGG-GA

SL-CL-DR_9-3 TTCAAAAA-TCGGT-CAAAAATGCCAAAA-ATG--GGTCCGGGGCGG-GA

SL-CL-DR_9-4 TTCAAAAA-TCGGT-CAAAAATGCCAAAA-ATG--GGTCCGGGGCGG-GA

SL-CL-DR_11-1 TTCAAAAA-TCGGT-CAAAAATGCCAAAA-ATC--GCTCCGGGGCGG-GA

SL-CL-DR_11-4 TTCAAAAA-TCGGT-CAAAAATGCCAAAA-ATC--GCTCCGGGGCGG-GA

SL-CL-DR_15-1 TTCAAAAA-TGGGT-CAAAAATGCCAAAA-ATG--GGTCCGGGGCGG-GA

SL-CL-DR_15-5 TTCAAAAA-TGGGT-CAAAAATGCCAAAA-ATG--GGTCCGGGGCGG-GA

SL-CL-DR_51-2 TTCAAAAA-TCGGT-CAAAAATCGCAAAA-ATG--GGTCCGGGGCGG-GA

SL-CL-DR_51-3 TTCAAAAA-TCGGT-CAAAAATCGCAAAA-ATG--GGTCCGGGGCGG-GA

SL-CL-DR_51-4 TTCAAAAA-TCGGT-CAAAAATCGCAAAA-ATG--GGTCCGGGGCGG-GA

SL-CL-DR_51-5 TTCAAAAA-TCGGT-CAAAAATCGCAAAA-ATG--GGTCCGGGGCGG-GA

SL-CL-DR_56-1 TTCAAAAA-TCGGT-CAAAAATGCCAAAA-ATG--GGTCCGGGGCGG-GA

SL-CL-DR_56-2 TTCAAAAA-TCGGT-CAAAAATGCCAAAA-ATG--GGTCCGGGGCGG-GA

SL-CL-DR_56-4 TTCAAAAA-TCGGT-CAAAAATGCCAAAA-ATG--GGTCCGGGGCGG-GA

SL-CL-DR_56-5 TTCAAAAA-TCGGT-CAAAAATGCCAAAA-ATG--GGTCCGGGGCGG-GA

SL-VL_52-1 TTGAAAAATTAGGC--AAAAATGCCAAAA-ATCG-GCTCCGAGGCGG-GA

SL-VL_52-2 TTGAAAAATTAGGC--AAAAATGCCAAAA-ATCG-GCTCCGAGGCGG-GA

SL-VL_52-3 TTGAAAAATTAGGC--AAAAATGCCAAAA-ATCG-GCTCCGAGGCGG-GA

SL-VL_52-4 TTGAAAAATTAGGC--AAAAATGCCAAAA-ATCG-GCTCCGAGGCGG-GA

SL-VL_52-5 TTGAAAAATTAGGC--AAAAATGCCAAAA-ATCG-GCTCCGAGGCGG-GA

SL-VL_52-6 TTGAAAAATTAGGC--AAAAATGCCAAAA-ATCG-GCTCCGAGGCGG-GA

SL-VL_53-1 TTCAAAAAATAGGC--AAAAATGCCAAAA-ATCG-GCTCCGAGGCGG-GA

SL-VL_53-2 TTCAAAAAATAGGC--AAAAATGCCAAAA-ATCG-GCTCCGAGGCGG-GA

SL-VL_53-3 TTCAAAAAATAGGC--AAAAATGCCAAAA-ATCG-GCTCCGAGGCGG-GA

SL-VL_53-4 TTCAAAAAATAGGC--AAAAATGCCAAAA-ATCG-GCTCCGAGGCGG-GA

SL-VL_53-5 TTCAAAAAATAGGC--AAAAATGCCAAAA-ATCG-GCTCCGAGGCGG-GA

SL-VL_54-1 TTCAAAAAATAGGC--AAAAATCGCAAAA-ATCG-GCTCCGAGGCGG-GA

SL-VL_54-2 TTCAAAAAATAGGC--AAAAATCGCAAAA-ATCG-GCTCCGAGGCGG-GA

SL-VL_54-3 TTCAAAAAATAGGC--AAAAATCGCAAAA-ATCG-GCTCCGAGGCGG-GA

SL-VL_54-4 TTCAAAAAATAGGC--AAAAATCGCAAAA-ATCG-GCTCCGAGGCGG-GA

SL-VL_54-5 TTCAAAAAATAGGC--AAAAATCGCAAAA-ATCG-GCTCCGAGGCGG-GA

SL-VL_55-1 TTCAAAAAATACGC--AAAAATGCCAAAA-ATCG-GCTCCGAGGCGG-GA

SL-VL_55-2 TTCAAAAAATACGC--AAAAATGCCAAAA-ATCG-GCTCCGAGGCGG-GA

SL-VL_55-4 TTCAAAAAATACGC--AAAAATGCCAAAA-ATCG-GCTCCGAGGCGG-GA

SL-VL_55-5 TTCAAAAAATACGC--AAAAATGCCAAAA-ATCG-GCTCCGAGGCGG-GA

SL-VL_55-6 TTCAAAAAATACGC--AAAAATGCCAAAA-ATCG-GCTCCGAGGCGG-GA

IN-CL_10-171 TTCATTTT-TTGGT-CAAAATCAGCATTT-TTGG-GCTCCGGGGTGG-AA

IN-CL_10-170 TTCATTTT-TTGGT-CAAAATCAGCATTT-TTGG-GCTCCGGGGTGG-AA

IN-CL_10-173 TTCATTTT-TTGGT-CAAAATCAGCATTT-TTGG-GCTCCGGGGTGG-AA

IN-CL_10-1731 TTCATTTT-TTGGT-CAAAATCAGCATTT-TTGG-GCTCCGGGGTGG-AA

IN-PKDL_83-166 TTCAAAAA-TCGGC-AGAAAATGCCAAAA-ATCG-GCTCCGAGGCGG-AA

IN-PKDL_83-165 TTCAAAAA-TCGGC-CGAAAATGCCAAAA-ATCA-GCTCCGGGGCGG-AA

IN-PKDL_83-168 TTCAAAAA-ATGGC-TAAAAATGCCAAAA-ATCG-GATTCGAGGCGG-GA

IN-VL_220-41 TTCAAAAA-ATGGC-CAAAAATGCCAAAA-ATCG-GCTCCGGGGCGG-GA

IN-VL_220-50 TTCAAAAA-ATGGC-CAAAAATGCCAAAA-ATCG-GCTCCGGGGCGG-GA

IN-VL_220-51 TTCAAAAA-ATGGC-CAAAAATGCCAAAA-ATCG-GCTCCGAGGCGG-GA

Ld_DD8_33 TTCAAAAA-TCGGC-GGAAAATGCCAAAA-ATCG-GCTCCGAGGCGG-GA

Ld_DD8_34 TTCAAAAA-TCTGC-CAAAAATGCCAAAA-ATCG-GGTCCGGGGCGG-AA

Ld_DD8_35 TTCAAAAA-ATAGC-CAAAAATGCCAAAA-ATCG-GCTCCGGGGCGG-GA

Ld_DD8_36 TTCAAAAAAT-AGC-CAAAAATGCCAAAA-ATCG-GCTCCGAGGCGG-GA

NP-PKDL_446-16 TTCATTT--TTGGCCGAAAATTGGCATTT-TTGG-GGTCGGAGGCTG-CA

NP-PKDL_466-3 TTCATTT--TTGGCCGAAAATTGGCATTT-TTGG-GGTCGGAGGCTG-CA

NP-PKDL_446-17 TTCATTT--TTGGCCGAAAATTGGCATTT-TTGG-GGTCGGAGGCTG-CA

NP-PKDL_446-19 TTCATTT--TTGGCCGAAAATTGGCATTT-TTGG-GGTCGGAGGCTG-CA

NP-VL_813-6 TTCAAAAA-ATGCC-CAAAAATGCCAATT-TTGG-CCTTCGGGGCGG-AA

NP-VL_813-2 TTCAAAAA-ATAGC-C-AAATTGGCATTT-TTGA-GCTCCGGGGCGG-GA

NP-VL_813-7 TTCAAAAA-ATGCC-CAAAAATGCCAATT-TTGG-CCTTCGGGGCGG-AA

Lm_A1_18 TTCATAAT-TTGAC-CAAAAATGCCAAAA-ATGG-GCTCGGAGGCGG-GA

Lm_A1_16 TTCATAAT-TTGGC-CAAAAATGCCAAAA-ATGG-CCTCGAGGGCGG-GA

Lm_A1_20 TTCATAAT-TTGGC-CAAAAATGCCAAAA-ATGG-GCTCGGAGGCGG-GA

Lm_A1_10 TTCATAAT-TTGGC-CAAAAATGCCAAAA-ATGG-GCTCGGAGGCGG-GA

Lb_P_59 TTCATAAT-TCAGG-GGAATTCCTCGAAA-ATCG-GCTCCG-GGCGG-G-

Lb_P_60 TTCATATT-TTGGG-GGAATTCCTCGAAA-ATCG-GCTCCGGGGCTC---

Lb_P_61 TTCATAAT-TCAGG-GGAAAACCTCGAAA-ATCG-GCTC-GGGGCGG-G-

Lb_P_81 TTCATATT-TTGGG-CGGAAATTCCGAAA-ATCG-GCTC-GGGGCGG-G-

Lb_P_80 TTCATAAT-TTGGC-GGGAAACCCCGAAA-TTCG-GCTCCG-GGCGG-G-

Lb_P_62 TTCATATT-TTGGG-CAAAAATGCCAGAT-TTCG-GCTC-GGGGCGT-G-

Li-TK-89 TTCAAAAA-TCGGC-CAAAAATGCCAAAA-ATCA-GCTCCGGGGCGG-GA

Li-TK-85 TTCAAAAA-TTGTC-CAAAAATGCCAAAA-ATCG-GCTCCGGGGCGG-GA

Li-TK-83 TTCAAAAA-TCGGC-CAAAAATGCCAAAA-ATCG-GCTCCGGGGCGG-GA

Li-TK-91 TTCAAAAA-TCGGC-CAAAAATGCCAAAA-ATCG-GCTCCGGGGCGG-GA

Li-TK-84 TTCAAAAA-TCGGC-CAAAAATGCCAAAA-ATCA-GCTCCGGGGCGG-GA

Li_SP_57 TTCATTTT-T-GGCTCGAAAATGCCATTT-TTGG-CCTCCGGGGCGG-GA

Li_SP_54 TTCATTTT-T-GGCCCGAAAATGCCATTT-TTGG-CCTCCGGGGCGG-GA

Li_SP_52 TTCATTTT-TTGGC-CAAAATCGCCATTT-TTGG-GGTCGGAGGCGG-AA

Li_SP_56 TTCATTTT-T-GGCCCGAAAATGCCATTT-TTGG-CCTCCGGGGCGG-GA

Li_SP_53 TTCATTTT-T-GGCCCGAAAATGCCATTT-TTGG-CCTCCGGGGCGG-GA

Li_SP_55 TTCATTTT-T-GGCCCGAAAATGCCATTT-TTGG-CCTCCGGGGCGG-GA

Lm_A1_9 TTCATTT--TTGGCTGGAAATTGCCATTT-TTGG-GCTCCGGGGCGG-GA

Lm_A1_7 TTCATTTT-TGGTC-GGAAAACGCCATTT-TTGG-GCTCGGAGGCGG-GA

Lm_A1_17 TTCATTTT-TTGTC-GAAAATTGGCATTT-TTGA-GCTCCGGGGCGG-GA

Lm_A1_19 TTCAAAAA-TCAGC-CAAAAATGCCGTTTTTTAG-GCCTCG-GGCGG-GA

Lt_TK_3 TTCAAAAA-TCCTGA-AAAAATGCCATTT-TTGG-GCTCGGAGGCTG-CA

Lt_TK_14 TTCAAAAA-TTGGCCA-AAAATGCCATT--TTCGGGGTCGGAGGCGT-CA

Lt_TK_5 TTCAAAAA-TTGGCCA-AAAATGCCATT--TTCGGGGTCGGAGGCGT-CA

Lam-322 TTCATATT-TTGAC-CAAAAATCCCGAAA-TTCG-GCTC--GGGCGG-TC

Lam-325 TTCATATT-TTTGG-GGAATTCGAGGAAT-TTCG-GCTCCGG--CGGTG-

Lam-323 TTCATATT-TTGGC-CAAAAATCTCGAAA-TTCG-GCTC--GGACGG-TC

Lam-331 TTCATAAT-TTGGC-CAAAAATCCTGGAA-ATCG-GCTC--GGGCGG-TC

* * *

SL-CL-S_2-1 --AACTGGGGGTTGGTGTAAAATAGG

SL-CL-S_2-6 --AACTGGGGGTTGGTGTAAAATAGG

SL-CL-S_2-7 --AACTGGGGGTTGGTGTAAAATAGG

SL-CL-S_2-8 --AACTGGGGGTTGGTGTAAAATAGG

SL-CL-S_2-9 --AACTGGGGGTTGGTGTAAAATAGG

SL-CL-S_2-10 --AACTGGGGGTTGGTGTAAAATAGG

SL-CL-S_3-2 --AACTGGGGGTTGGTGTAAAATAGG

SL-CL-S_3-4 --AACTGGGGGTTGGTGTAAAATAGG

SL-CL-S_3-5 --AACTGGGGGTTGGTGTAAAATAGG

SL-CL-S_3-7 --AACTGGGGGTTGGTGTAAAATAGG

SL-CL-S_3-8 --AACTGGGGGTTGGTGTAAAATAGG

SL-CL-S_3-9 --AACTGGGGGTTGGTGTAAAATAGG

SL-CL-S_6-3 --AACTGGGGGTTGGTGTAAAATAGG

SL-CL-S_6-5 --AACTGGGGGTTGGTGTAAAATAGG

SL-CL-S_6-6 --AACTGGGGGTTGGTGTAAAATAGG

SL-CL-S_6-2 --AACTGGGGGTTGGTGTAAAATAGG

SL-CL-S_7-2 --AACTGGGGGTTGGTGTAAAATAGG

SL-CL-S_7-6 --AACTGGGGGTTGGTGTAAAATAGG

SL-CL-S_7-7 --AACTGGGGGTTGGTGTAAAATAGG

SL-CL-S_7-8 --AACTGGGGGTTGGTGTAAAATAGG

SL-CL-S_8-3 --AACTGGGGGTTGGTGTAAAATAGG

SL-CL-S_8-4 --AACTGGGGGTTGGTGTAAAATAGG

SL-CL-S_8-5 --AACTGGGGGTTGGTGTAAAATAGG

SL-CL-S_8-6 --AACTGGGGGTTGGTGTAAAATAGG

SL-CL-S_10-1 --AACTGGGGGTTGGTGTAAAATAGG

SL-CL-S_10-2 --AACTGGGGGTTGGTGTAAAATAGG

SL-CL-S_10-3 --AACTGGGGGTTGGTGTAAAATAGG

SL-CL-S_12-1 --AACTGGGGGTTGGTGTAAAATAGG

SL-CL-S_12-2 --AACTGGGGGTTGGTGTAAAATAGG

SL-CL-S_12-3 --AACTGGGGGTTGGTGTAAAATAGG

SL-CL-S_13-1 --AACTGGGGGTTGGTGTAAAATAGG

SL-CL-S_13-2 --AACTGGGGGTTGGTGTAAAATAGG

SL-CL-S_13-3 --AACTGGGGGTTGGTGTAAAATAGG

SL-CL-S_14-1 --AACTGGGGGTTGGTGTAAAATAGG

SL-CL-S_14-2 --AACTGGGGGTTGGTGTAAAATAGG

SL-CL-S_14-3 --AACTGGGGGTTGGTGTAAAATAGG

SL-CL-S_16-3 --AACTGGGGGTTGGTGTAAAATAGG

SL-CL-S_16-4 --AACTGGGGGTTGGTGTAAAATAGG

SL-CL-S_16-6 --AACTGGGGGTTGGTGTAAAATAGG

SL-CL-S_31-1 --AACTGGGGGTTGGTGTAAAATAGG

SL-CL-S_31-2 --AACTGGGGGTTGGTGTAAAATAGG

SL-CL-S_31-3 --AACTGGGGGTTGGTGTAAAATAGG

SL-CL-S_31-4 --AACTGGGGGTTGGTGTAAAATAGG

SL-CL-S_31-5 --AACTGGGGGTTGGTGTAAAATAGG

SL-CL-S_32-1 --AACTGGGGGTTGGTGTAAAATAGG

SL-CL-S_32-2 --AACTGGGGGTTGGTGTAAAATAGG

SL-CL-S_32-3 --AACTGGGGGTTGGTGTAAAATAGG

SL-CL-S_32-4 --AACTGGGGGTTGGTGTAAAATAGG

SL-CL-S_32-5 --AACTGGGGGTTGGTGTAAAATAGG

SL-CL-S_33-1 --AACTGGGGGTTGGTGTAAAATAGG

SL-CL-S_33-2 --AACTGGGGGTTGGTGTAAAATAGG

SL-CL-S_33-3 --AACTGGGGGTTGGTGTAAAATAGG

SL-CL-S_33-4 --AACTGGGGGTTGGTGTAAAATAGG

SL-CL-S_33-5 --AACTGGGGGTTGGTGTAAAATAGG

SL-CL-S_34-1 --AACTGGGGGTTGGTGTAAAATAGG

SL-CL-S_34-2 --AACTGGGGGTTGGTGTAAAATAGG

SL-CL-S_34-3 --AACTGGGGGTTGGTGTAAAATAGG

SL-CL-S_34-4 --AACTGGGGGTTGGTGTAAAATAGG

SL-CL-S_34-5 --AACTGGGGGTTGGTGTAAAATAGG

SL-CL-S_35-1 --AACTGGGGGTTGGTGTAAAATAGG

SL-CL-S_35-3 --AACTGGGGGTTGGTGTAAAATAGG

SL-CL-S_35-4 --AACTGGGGGTTGGTGTAAAATAGG

SL-CL-S_35-6 --AACTGGGGGTTGGTGTAAAATAGG

SL-CL-S_36-1 --AACTGGGGGTTGGTGTAAAATAGG

SL-CL-S_36-2 --AACTGGGGGTTGGTGTAAAATAGG

SL-CL-S_36-3 --AACTGGGGGTTGGTGTAAAATAGG

SL-CL-S_36-4 --AACTGGGGGTTGGTGTAAAATAGG

SL-CL-S_37-1 --AACTGGGGGTTGGTGTAAAATAGG

SL-CL-S_37-2 --AACTGGGGGTTGGTGTAAAATAGG

SL-CL-S_37-3 --AACTGGGGGTTGGTGTAAAATAGG

SL-CL-S_37-4 --AACTGGGGGTTGGTGTAAAATAGG

SL-CL-S_38-1 --AACTGGGGGTTGGTGTAAAATAGG

SL-CL-S_38-2 --AACTGGGGGTTGGTGTAAAATAGG

SL-CL-S_38-3 --AACTGGGGGTTGGTGTAAAATAGG

SL-CL-S_38-4 --AACTGGGGGTTGGTGTAAAATAGG

SL-CL-S_38-5 --AACTGGGGGTTGGTGTAAAATAGG

SL-CL-S_39-1 --AACTGGGGGTTGGTGTAAAATAGG

SL-CL-S_39-2 --AACTGGGGGTTGGTGTAAAATAGG

SL-CL-S_39-3 --AACTGGGGGTTGGTGTAAAATAGG

SL-CL-S_39-4 --AACTGGGGGTTGGTGTAAAATAGG

SL-CL-S_39-5 --AACTGGGGGTTGGTGTAAAATAGG

SL-CL-S_40-1 --AACTGGGGGTTGGTGTAAAATAGG

SL-CL-S_40-3 --AACTGGGGGTTGGTGTAAAATAGG

SL-CL-S_40-4 --AACTGGGGGTTGGTGTAAAATAGG

SL-CL-S_40-5 --AACTGGGGGTTGGTGTAAAATAGG

SL-CL-S_41-1 --AACTGGGGGTTGGTGTAAAATAGG

SL-CL-S_41-2 --AACTGGGGGTTGGTGTAAAATAGG

SL-CL-S_41-4 --AACTGGGGGTTGGTGTAAAATAGG

SL-CL-S_41-5 --AACTGGGGGTTGGTGTAAAATAGG

SL-CL-S_42-2 --AACTGGGGGTTGGTGTAAAATAGG

SL-CL-S_42-3 --AACTGGGGGTTGGTGTAAAATAGG

SL-CL-S_42-5 --AACTGGGGGTTGGTGTAAAATAGG

SL-CL-S_43-1 --AACTGGGGGTTGGTGTAAAATAGG

SL-CL-S_43-3 --AACTGGGGGTTGGTGTAAAATAGG

SL-CL-S_43-4 --AACTGGGGGTTGGTGTAAAATAGG

SL-CL-S_43-5 --AACTGGGGGTTGGTGTAAAATAGG

SL-CL-S_43-6 --AACTGGGGGTTGGTGTAAAATAGG

SL-CL-S_44-1 --AACTGGGGGTTGGTGTAAAATAGG

SL-CL-S_44-2 --AACTGGGGGTTGGTGTAAAATAGG

SL-CL-S_44-3 --AACTGGGGGTTGGTGTAAAATAGG

SL-CL-S_44-6 --AACTGGGGGTTGGTGTAAAATAGG

SL-CL-S_45-3 --AACTGGGGGTTGGTGTAAAATAGG

SL-CL-S_46-1 --AACTGGGGGTTGGTGTAAAATAGG

SL-CL-S_46-2 --AACTGGGGGTTGGTGTAAAATAGG

SL-CL-S_46-4 --AACTGGGGGTTGGTGTAAAATAGG

SL-CL-S_46-6 --AACTGGGGGTTGGTGTAAAATAGG

SL-CL-S_48-1 --AACTGGGGGTTGGTGTAAAATAGG

SL-CL-S_48-3 --AACTGGGGGTTGGTGTAAAATAGG

SL-CL-S_49-4 --AACTGGGGGTTGGTGTAAAATAGG

SL-CL-S_49-5 --AACTGGGGGTTGGTGTAAAATAGG

SL-CL-S_50-1 --AACTGGGGGTTGGTGTAAAATAGG

SL-CL-S_50-4 --AACTGGGGGTTGGTGTAAAATAGG

SL-CL-S_50-6 --AACTGGGGGTTGGTGTAAAATAGG

SL-CL-DR_1-2 -AAACTGGGGGTTGGTGTAAAATAGG

SL-CL-DR_1-4 -AAACTGGGGGTTGGTGTAAAATAGG

SL-CL-DR_1-6 -AAACTGGGGGTTGGTGTAAAATAGG

SL-CL-DR_1-7 -AAACTGGGGGTTGGTGTAAAATAGG

SL-CL-DR_1-8 -AAACTGGGGGTTGGTGTAAAATAGG

SL-CL-DR_1-9 -AAACTGGGGGTTGGTGTAAAATAGG

SL-CL-DR_1-10 -AAACTGGGGGTTGGTGTAAAATAGG

SL-CL-DR_9-1 -AAACTGGGGGTTGGTGTAAAATAGG

SL-CL-DR_9-2 -AAACTGGGGGTTGGTGTAAAATAGG

SL-CL-DR_9-3 -AAACTGGGGGTTGGTGTAAAATAGG

SL-CL-DR_9-4 -AAACTGGGGGTTGGTGTAAAATAGG

SL-CL-DR_11-1 -AAACTGGGGGTTGGTGTAAAATAGG

SL-CL-DR_11-4 -AAACTGGGGGTTGGTGTAAAATAGG

SL-CL-DR_15-1 -AAACTGGGGGTTGGTGTAAAATAGG

SL-CL-DR_15-5 -AAACTGGGGGTTGGTGTAAAATAGG

SL-CL-DR_51-2 -AAACTGGGGGTTGGTGTAAAATAGG

SL-CL-DR_51-3 -AAACTGGGGGTTGGTGTAAAATAGG

SL-CL-DR_51-4 -AAACTGGGGGTTGGTGTAAAATAGG

SL-CL-DR_51-5 -AAACTGGGGGTTGGTGTAAAATAGG

SL-CL-DR_56-1 -AAACTGGGGGTTGGTGTAAAATAGG

SL-CL-DR_56-2 -AAACTGGGGGTTGGTGTAAAATAGG

SL-CL-DR_56-4 -AAACTGGGGGTTGGTGTAAAATAGG

SL-CL-DR_56-5 -AAACTGGGGGTTGGTGTAAAATAGG

SL-VL_52-1 --AACTGGGGGTTGGTGTAAAATAGG

SL-VL_52-2 --AACTGGGGGTTGGTGTAAAATAGG

SL-VL_52-3 --AACTGGGGGTTGGTGTAAAATAGG

SL-VL_52-4 --AACTGGGGGTTGGTGTAAAATAGG

SL-VL_52-5 --AACTGGGGGTTGGTGTAAAATAGG

SL-VL_52-6 --AACTGGGGGTTGGTGTAAAATAGG

SL-VL_53-1 --AACTGGGGGTTGGTGTAAAATAGG

SL-VL_53-2 --AACTGGGGGTTGGTGTAAAATAGG

SL-VL_53-3 --AACTGGGGGTTGGTGTAAAATAGG

SL-VL_53-4 --AACTGGGGGTTGGTGTAAAATAGG

SL-VL_53-5 --AACTGGGGGTTGGTGTAAAATAGG

SL-VL_54-1 --AACTGGGGGTTGGTGTAAAATAGG

SL-VL_54-2 --AACTGGGGGTTGGTGTAAAATAGG

SL-VL_54-3 --AACTGGGGGTTGGTGTAAAATAGG

SL-VL_54-4 --AACTGGGGGTTGGTGTAAAATAGG

SL-VL_54-5 --AACTGGGGGTTGGTGTAAAATAGG

SL-VL_55-1 --AACTGGGGGTTGGTGTAAAATAGG

SL-VL_55-2 --AACTGGGGGTTGGTGTAAAATAGG

SL-VL_55-4 --AACTGGGGGTTGGTGTAAAATAGG

SL-VL_55-5 --AACTGGGGGTTGGTGTAAAATAGG

SL-VL_55-6 --AACTGGGGGTTGGTGTAAAATAGG

IN-CL_10-171 --AACTGGGGGTTGGTGTAAAATAGG

IN-CL_10-170 --AACTGGGGGTTGGTGTAAAATAGG

IN-CL_10-173 --AACTGGGGGTTGGTGTAAAATAGG

IN-CL_10-1731 --AACTGGGGGTTGGTGTAAAATAGG

IN-PKDL_83-166 --AACTGGGGGTTGGTGTAAAATAGG

IN-PKDL_83-165 --AACTGGGGGTTGGTGTAAAATAGG

IN-PKDL_83-168 --AACTGGGGGTTGGTGTAAAATAGG

IN-VL_220-41 --AACTGGGGGTTGGTGTAAAATAGG

IN-VL_220-50 --AACTGGGGGTTGGTGTAAAATAGG

IN-VL_220-51 --AACTGGGGGTTGGTGTAAAATAGG

Ld_DD8_33 --AACTGGGGGTTGGTGTAAAATAGG

Ld_DD8_34 --AACTGGGGGTTGGTGTAAAATAGG

Ld_DD8_35 --AACTGGGGGTTGGTGTAAAATAGG

Ld_DD8_36 --AACTGGGGGTTGGTGTAAAATAGG

NP-PKDL_446-16 --AACTGGGGGTTGGTGTAAAATAGG

NP-PKDL_466-3 --AACTGGGGGTTGGTGTAAAATAGG

NP-PKDL_446-17 --AACTGGGGGTTGGTGTAAAATAGG

NP-PKDL_446-19 --AACTGGGGGTTGGTGTAAAATAGG

NP-VL_813-6 --AACTGGGGGTTGGTGAATCACTAG

NP-VL_813-2 -AAACTGGGGGTTGGTGTAAAATAGG

NP-VL_813-7 --AACTGGGGGTTGGTGAATCACTAG

Lm_A1_18 --AACTGGGGGTTGGTGTAAAATAGG

Lm_A1_16 --AACTGGGGGTTGGTGTAAAATAGG

Lm_A1_20 --AACTGGGGGTTGGTGTAAAATAGG

Lm_A1_10 --AACTGGGGGTTGGTGTAAAATAGG

Lb_P_59 GAAACTGGGGGTTGGTGTAAAATAGG

Lb_P_60 GAAACTGGGGGTTGGTGTAAAATAGG

Lb_P_61 GAAACTGGGGGTTGGTGTAAAATAGG

Lb_P_81 GAAACTGGGGGTTGGTGTAAAATAGG

Lb_P_80 GAAACTGGGGGTTGGTGTAAAATAGG

Lb_P_62 GAAACTGGGGGTTGGTGTAAAATAGG

Li-TK-89 --AACTGGGGGTTGGTGTAAAATAGG

Li-TK-85 --AACTGGGGGTTGGTGTAAAATAGG

Li-TK-83 --AACTGGGGGTTGGTGTAAAATAGG

Li-TK-91 --AACTGGGGGTTGGTGTAAAATAGG

Li-TK-84 --AACTGGGGGTTGGTGTAAAATAGG

Li_SP_57 --AACTGGGGGTTGGTGTAAAATAGG

Li_SP_54 --AACTGGGGGTTGGTGTAAAATAGG

Li_SP_52 --AACTGGGGGTTGGTGTAAAATAGG

Li_SP_56 --AACTGGGGGTTGGTGTAAAATAGG

Li_SP_53 --AACTGGGGGTTGGTGTAAAATAGG

Li_SP_55 --AACTGGGGGTTGGTGTAAAATAGG

Lm_A1_9 --AACTGGGGGTTGGTGTAAAATAGG

Lm_A1_7 --AACTGGGGGTTGGTGTAAAATAGG

Lm_A1_17 --AACTGGGGGTTGGTGTAAAATAGG

Lm_A1_19 --AACTGGGGGTTGGTGTAAAATAGG

Lt_TK_3 --AACTGGGGGTTGGTGTAAAATAGG

Lt_TK_14 --CTCTGGGGGTTGGTGTAAAATAGG

Lt_TK_5 --CTCTGGGGGTTGGTGTAAAATAGG

Lam-322 ACAACTGGGGGTTGGTGTAAAATAGG

Lam-325 AAAACTGGGGGTTGGTGTAAAATAGG

Lam-323 ACAACTGGGGGTTGGTGTAAAATAGG

Lam-331 ACAACTGGGGGTTGGTGTAAAATAGG

************* * * *

**Additional file 1: Multiple sequence alignment**

Multiple sequence alignment of the Sri Lankan, Indian and Nepalese isolates along with the reference sequences using CLUSTAL 2.1 multiple sequence alignment. Dashes(-) represent gaps introduced to optimize alignment. Asteriks (*) represent consensus nucleotides in the sequence. Lb(P):*L. braziliensis* [Peru] (Skin);Ld(DD8)/Ld(IN):*L. donovani* [India] (Bone marrow); Li(SP):*L. infantum* [Spain] (Unknown); Lm(A1):*L. major* [Libya]; Lt(TK):*L. tropica* [Turkey] (Skin); Lam: *L. amazonenis* (Skin); Li(TK):*L. infantum* [Turkey] (Spleen); SL-VL (52-55): VL Sri Lankan isolate (Bone marrow); SL_CL-S:*Leishmania* CL Sri Lankan isolate (Skin); SL_CL-DR:*Leishmania* CL Sri Lankan that failed to respond to antimonial drugs (Skin); IN-VL: *L. donovani* [India] (Bone marrow); IN-PKDL: *Leishmania* from post-kala azar dermal leishmaniasis (PKDL) [India] (Skin); NP-VL: *Leishmania* VL Nepalian isolate (Bone marrow); NP-PKDL: *Leishmania* cause PKDL [Nepal] (Skin).
