# Supplementary material for: Genetic diversity of Leishmania donovani that causes cutaneous leishmaniasis in Sri Lanka: a cross sectional study with regional comparisons
Source: BMC Infect Dis. 2017 Dec 22;17:791. doi: 10.1186/s12879-017-2883-x (PMC5741890; doi:10.1186/s12879-017-2883-x)
Supplement: Supplementary file 2 — The isolation by distance analysis showed the linear correlation between genetic diversity and geographic distance. Three populations were defined such as Sri Lanka, India and Nepal. However, this correlation was not statistically significant. (DOC 175 kb) [file 12879_2017_2883_MOESM2_ESM.doc]

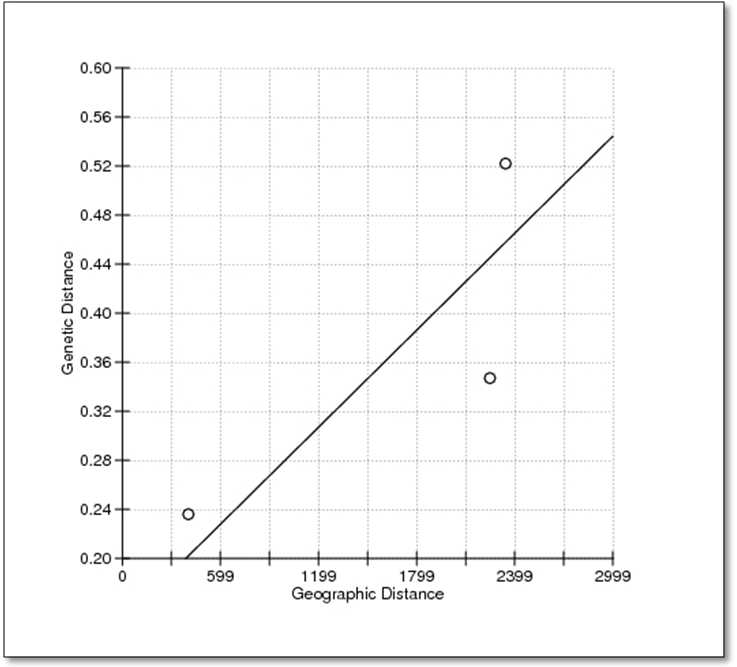


**Additional file 2:** The isolation by distance analysis showed the linear correlation between genetic diversity and geographic distance. Three populations were defined such as Sri Lanka, India and Nepal. However, this correlation was not statistically significant.
